# Tunable Core–Shell Metal Alloy Pillar Design in Vertically Aligned Nanostructures Toward Multifunctionality

**DOI:** 10.1002/smsc.202500622

**Published:** 2026-03-19

**Authors:** Abhijeet Choudhury, Benson Kunhung Tsai, Ping Lu, D. Hermawan, Lizabeth Quigley, Jialong Huang, Zedong Hu, Jeremy Gan, Natalia Garcia Godinez, James P. Barnard, Bharat Giri, A. Sanjuan, César Martínez‐Sánchez, Xiaoshan Xu, R. Edwin García, Haiyan Wang

**Affiliations:** ^1^ School of Materials Engineering Purdue University West Lafayette Indiana United States; ^2^ Sandia National Laboratories Albuquerque New Mexico USA; ^3^ Center for Integrated Nanotechnologies Sandia National Laboratories Albuquerque New Mexico USA; ^4^ School of Electrical and Computer Engineering Purdue University West Lafayette Indiana United States; ^5^ Department of Physics and Astronomy University of Nebraska Lincoln Nebraska USA; ^6^ Institute of Physics Universidad Nacional Autónoma de México Mexico City Mexico

**Keywords:** core–shell alloy nanopillar, magnetic anisotropy, nanoscale metal phase separation, optical anisotropy, vertically aligned nanocomposite

## Abstract

Multiphase nanostructures provide a powerful platform for multifunctional materials by integrating different phases with unique functionalities for coupling effects. Here, we explore an alloy‐based vertically aligned nanocomposite (VAN) thin films consisting of Au, Cu, Co, and Ni alloy nanopillars embedded in a BaTiO_3_ (BTO) matrix. Core–shell alloy VANs with tunable pillar morphology, ranging from spherical to faceted shapes, are fabricated by pulsed laser deposition by controlling substrate temperature and deposition frequency. These multiphase systems demonstrate tailorable optical anisotropy, tunable hyperbolic response, strong magnetic anisotropy, plasmonic resonances, and magneto‐optical coupling, highlighting their potential for spintronic and photonic applications. Calculations show pillar shape morphology is a result of the interplay of surface energies, epitaxial strain, and thermal expansion mismatch between the substrate and the VAN, while spinodal decomposition kinetics of the alloy defines a time–temperature–transformation diagram for controlling the morphology inside the pillar. This work gives insight into the dynamics of phase separation and structure–property relationships in complex alloy VANs allowing for design of next‐generation hybrid metamaterials.

## Graphical abstract

Core–shell alloy‐based BaTiO_3_/Au–Cu–Co–Ni vertically aligned nanocomposites exhibit tunable nanopillar morphologies governed by interfacial energetics. A temperature–time–transformation diagram predicts nanopillar evolution, while structure–property relationships link morphology with controllable optical anisotropy, hyperbolic dispersion, magnetic anisotropy, and magneto‐optical coupling, establishing a multifunctional platform for spintronic and photonic devices.

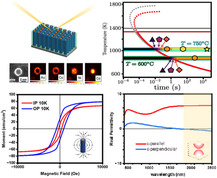

## 1 Introduction

Engineered nanostructures with diverse functionalities, such as magnetic, optical, and electrical behavior, fuel the development of next generation devices [1, 2, 3, 4, 5, 6, 7]. For aiding the efficient fabrication of advanced devices, multiphase films integrated with multifunctionalities present significant advantages over traditional single‐phase films. Among them, vertically aligned nanocomposite (VAN) thin films, which generally consist of two or more epitaxially grown phases, are in a unique position considering their structural anisotropy, multifunctionality, and interfacial coupling of properties throughout the film thickness [8, 9, 10, 11]. VAN films consist of self‐assembled nanopillars as the secondary phase in a matrix made of dielectric oxides or transition metal nitrides, which is called the primary phase. This creates an interesting vertical architecture resulting in a large vertical interfacial area between two different phases [12]. The resulting three‐dimensional strain distribution throughout the entire film allows for more precise tunability in the properties by manipulation of the microstructure of the secondary nanostructures, as compared to a multilayered film having only strain induced in‐plane resulting in a thickness limitation [13].

Most of the VAN thin films were deposited using pulsed laser deposition (PLD) to achieve high film crystallinity and epitaxial quality. Compared to the typical top‐down approaches for nanoscale multifunctional nanostructures, such as etching, lithography, and patterning, or bottom‐up methods such as templates, VAN growth can be completed in one step growth which is simpler and more cost‐effective [14]. Since the initial demonstration of VAN fabrication in oxide–oxide systems, the scope of VAN architectures has significantly expanded to include oxide–metal VANs, nitride–metal VANs, and complex multiphase systems, allowing for harnessing the unique properties of various material systems [15, 16, 17, 18, 19]. Especially, the incorporation of metals in the VAN systems, allowing for growth of metallic nanopillars in an oxide matrix, which adds more functionalities while increasing anisotropy in the film. Some of the substantial examples include incorporation of plasmonic metal nanopillars, such as Au, Cu, and Ag which introduce surface plasmon resonance (SPR) and optical anisotropy, while the ferromagnetic metal nanopillars such as Co, Fe, and Ni, allow for introducing magnetic anisotropy [20, 21, 22, 23, 24, 25]. However, despite various single metal incorporation in oxide and nitride matrixes as VANs, there have been very limited demonstrations of multielements metal alloys in VANs due to the contrasting growth kinetics and material behaviors of metals and oxides/nitrides [26, 27, 28].

From the fundamental growth mechanism aspect, the fabrication of multielemental metal alloy VANs presents valuable opportunities for investigating the interplay between kinetics and thermodynamics in VAN thin film systems and thus provides critical insights into phase separation behavior at the nanoscale. The two factors can cooperate or compete to give rise to unique structures such as core‐shell, Janus‐particle‐like (i.e., two‐sided particle structure), or even clustered domains. Thus, they serve as unique testing platforms to study the impact of deposition parameters such as deposition rate (e.g., laser frequency) and substrate temperature on the pillar morphology [29, 30, 31]. Thus, understanding the processing‐structure‐property correlation of multielement metal VANs is crucial for designing the multifunctional nanomaterials suitable for integration in next‐generational devices. Establishing a synthesis‐to‐property correlation is expected to provide a provide a guideline for optimizing performance toward specific functions, consistent with trends reported in earlier alloy studies [32, 33].

In this work, we have fabricated a series of metal alloy VANs under different deposition conditions, where barium titanate (BaTiO_3_, BTO) has been used as the dielectric matrix while the metal alloy consists of plasmonic metals (Au, Cu) and ferromagnetic metals (Co, Ni). The BTO matrix is selected because of its favorable lattice match with the strontium titanate (SrTiO_3_, STO) substrate, along with its excellent thermal stability and chemical inertness. The 4 metals (i.e., Au, Cu, Co, Ni in this case) were selected to integrate plasmonic and magnetic functionalities while enabling a controlled core–shell architecture within the nanopillars. Au is a noble plasmonic metal that should preferentially segregate to the outer surface due to its low surface energy among the four elements while forming an outer shell that is chemically inert. Cu is a plasmonic nonnoble metal that broadens the accessible design space while enabling plasmonic tuning. Co and Ni were selected to incorporate magnetic functionality, where the incorporation of Ni stabilizes Co in the fcc phase (vs hcp) through alloying effects. Furthermore, the immiscibility of the matrix with the metals allows for study of the phase separation at nanoscale, and various inherent properties enhance the overall functionality of the film [34, 35]. To investigate the influence of growth conditions, six films were deposited at two substrate temperatures, 600°C and 750°C, using the laser frequency of 2, 5, and 10 Hz. The microstructure characterization of all the films deposited has been carried out along with investigations into the optical and magnetic properties of the films, which reveal the structural‐property relationship in the films. These studies demonstrate the tunability of the VAN architecture through deposition parameters, enabling control over morphology of the VAN system. The findings could also offer critical insights into designing alloy VAN platforms for multifunctional applications.

## 2 Results and Discussion

**FIGURE 1:**
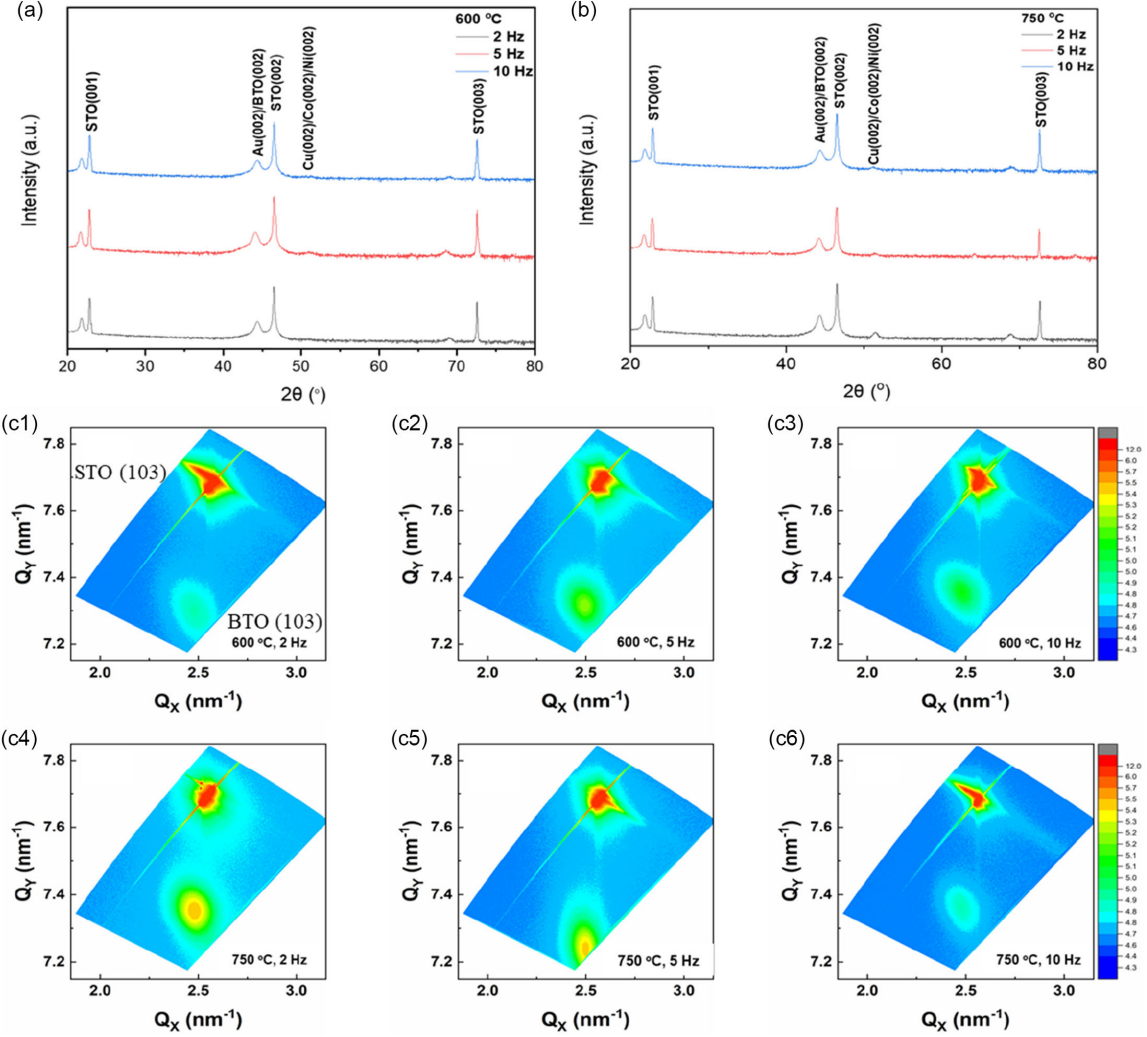
(a) *θ*‐2*θ* XRD scans of the alloy VAN films at 600°C (b) *θ*‐2*θ* XRD scans of the alloy VAN films at 750°C (c) RSM scans around the (103) peak of STO for the alloy VAN films.

A total of 6 BTO‐AuCuCoNi VAN thin films were deposited using two substrate temperatures (600°C and 750°C) and three laser deposition frequency (2, 5, and 10 Hz). In the following discussion, each sample is identified by its deposition temperature and laser frequency (e.g., ‘600°C, 10 Hz’). The films were first characterized using X‐ray diffraction (including *θ*–2*θ* scans and reciprocal space mapping (RSM)) for a comprehensive structural evaluation of the oxide alloy metal VAN films under the different deposition conditions (i.e., 600°C and 750°C at 2, 5, and 10 Hz) as shown in Figure 1. The *θ*–2*θ* XRD scans have confirmed the BTO (*00l*) peaks across all the films, which show the good (*00l*) texture of the BTO matrix with the substrate. The alloy peaks have not been detected in the scans, which could be attributed to the small size of the nanopillars, as previously reported, where only the BTO and sharp substrate (STO) peaks are observed [27]. No other secondary phases were identified, which suggests that there is no obvious reaction or interdiffusion between the BTO matrix and the metal alloy phases. The out‐of‐plane (OP) lattice parameter of BTO ranges in 4.08–4.10 Å across different deposition conditions (Table S5), and is slightly larger than the bulk phase (4.03 Å) [36], indicating OP tensile strain across all the nanocomposite samples. The in‐plane lattice parameter is in the range of 3.96–3.97 Å and is slightly less than that of the bulk BTO (3.99 Å) and is larger than that of the STO substrate (3.905 Å). The RSM characterization was conducted on all the samples around (103) asymmetric peak for getting more insight into the strain state in the film. All the RSMs show a sharply defined, high‐intensity peak on the upper side, which corresponds to the STO substrate (103), while a broader and lower intensity peak, corresponding to the BTO (103), is observed at lower Q_*x*_ and Q_*y*_ relative to the substrate peak. The plots show a shift in Q_*x*_ from the substrate, which confirms that the films do not have a pseudomorphic nature. The result also suggests that the BTO matrix has partially relaxed in‐plane from the STO compressive strain (*a* = 3.905 Å) [37], which could be attributed to the presence of oxygen vacancies and secondary metal alloy phases. The lower Q_*x*_ value of the BTO peak in comparison with STO peak corresponds to the expanded in‐plane lattice parameter *a* = 3.96–3.97 Å, while the lower Q_*y*_ in comparison with STO corresponds to the expanded OP parameter *c* = 4.08–4.10 Å. The broader and more diffuse nature of the film (BTO) peak compared to the STO peak in all the films is consistent with a complex strain distribution and possible mosaicity [38]. Notably, when comparing films grown under different temperatures, the BTO peaks for films deposited at 750°C are generally narrower than those grown at 600°C (Table S5), indicating improved epitaxial quality at higher growth temperature, which may be associated with enhanced adatom mobility during deposition.

**FIGURE 2:**
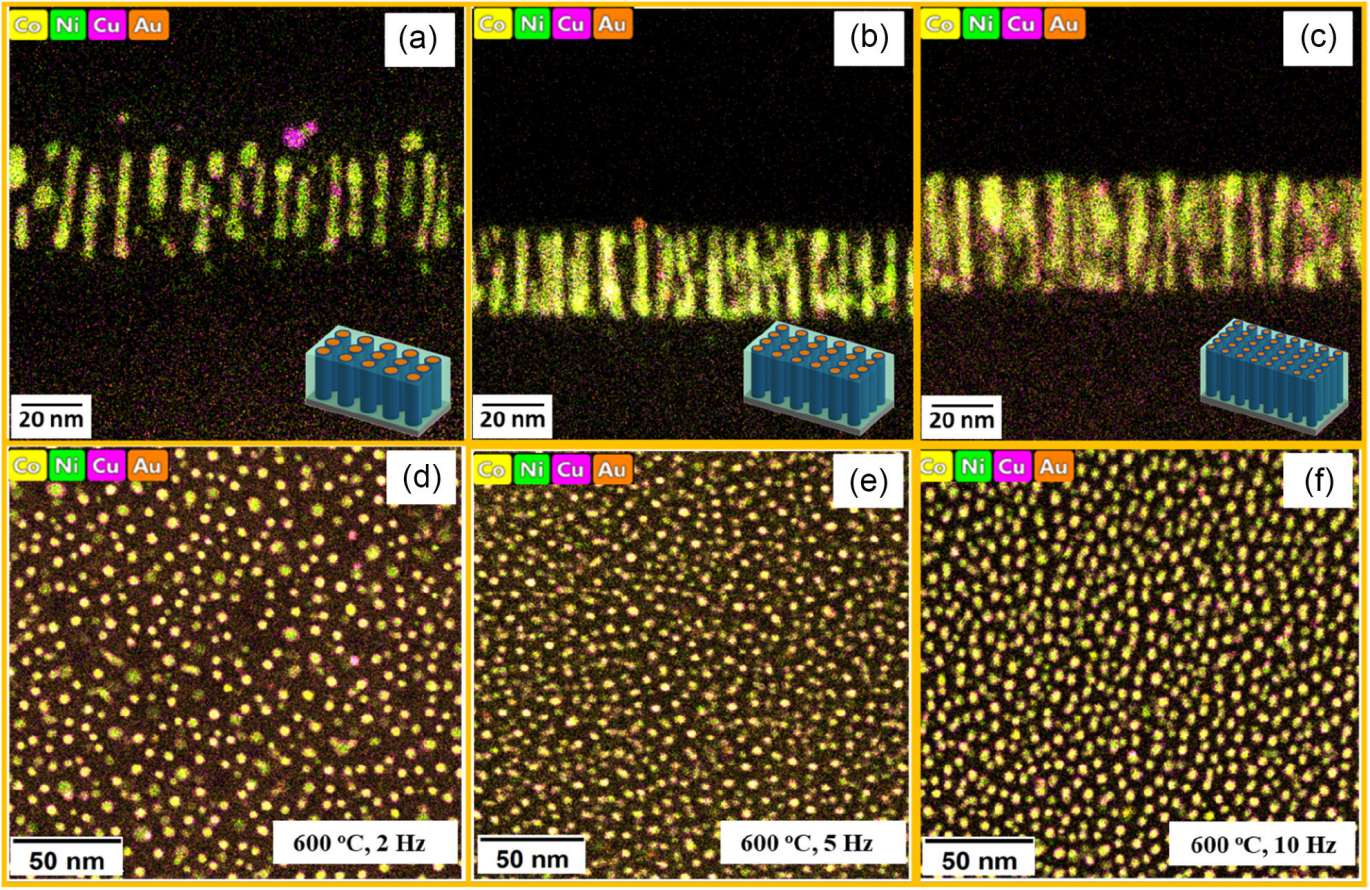
Cross‐section EDS maps of all the alloy VAN films deposited at 600°C with the schematics shown in the inset having deposition frequencies of (a) 2 Hz, (b) 5 Hz, and (c) 10 Hz. The corresponding plan‐view images are shown in (d) 2 Hz, (e) 5 Hz, and (f) 10 Hz.

**FIGURE 3:**
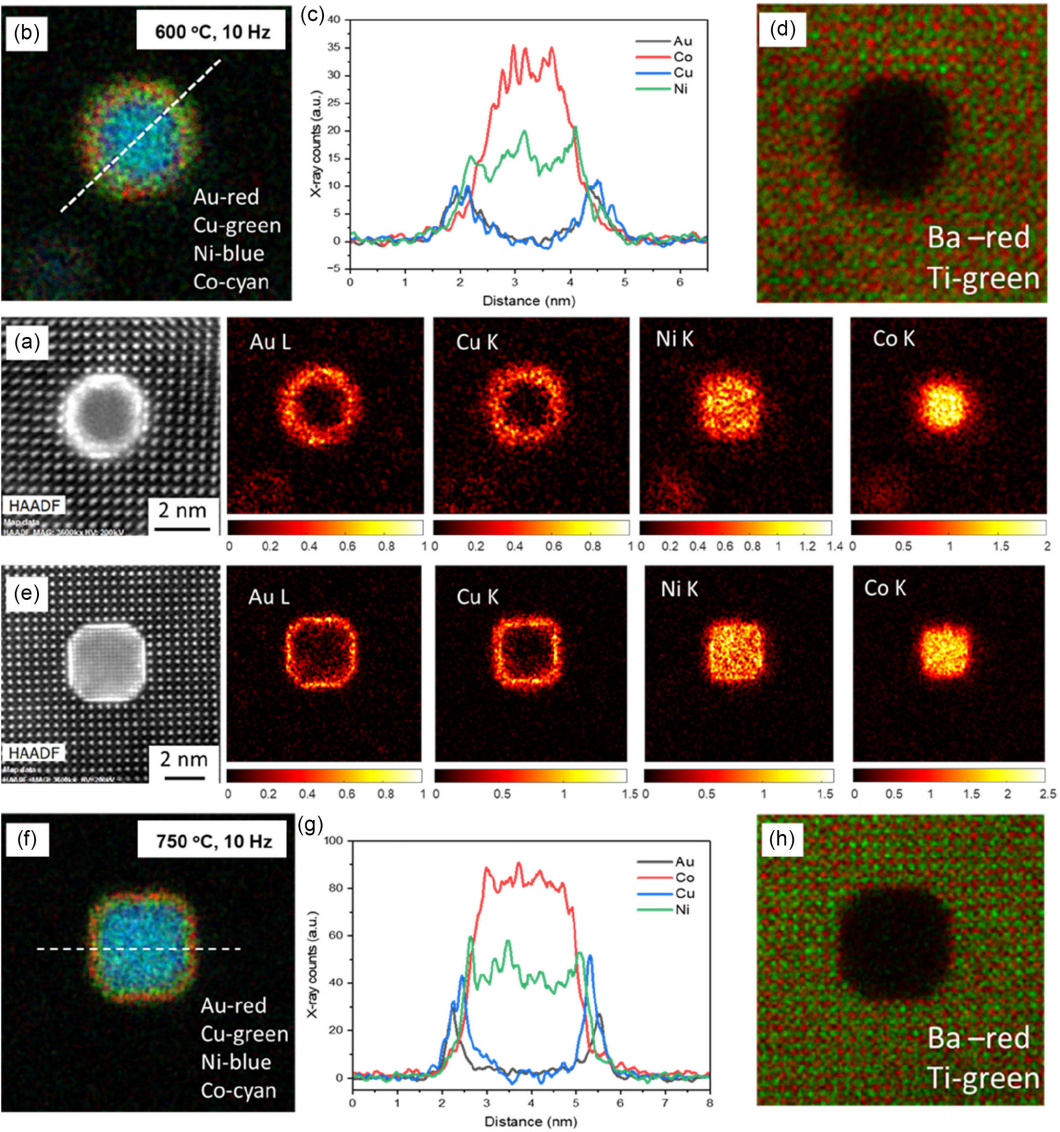
(a) High‐resolution STEM image of the alloy pillar at 600°C with the right panel showing the EDS map of individual elements, (b) EDS composite map consisting of Au, Cu, Ni, and Co with corresponding (c) line‐profiles of the elements and (d) EDS composite map of the matrix of the 600°C system (e) High‐resolution STEM image of the alloy pillar at 750°C with the right panel showing the EDS map of individual elements (f) EDS composite map consisting of Au, Cu, Ni, and Co with corresponding (g) line‐profiles of the elements and (h) and EDS composite map of the matrix of the 750°C system.

The microstructure of the alloy VAN films was further characterized by scanning transmission electron microscopy (STEM). The cross‐section (CS) energy‐dspersive spectroscopy (EDS) maps along with its plan‐view (PV) images of all the films deposited at 600°C are shown in Figure 2. The corresponding STEM images and individual elemental mappings are shown from Figure S1–S6. Similarly, the CS EDS maps, along with its plan‐view images of the films at 750°C accompanied by their STEM images and individual elemental mappings, are shown from Figure S7–S13. All the films exhibit a thickness of 30–40 nm. From the figures, we can observe that the metal alloy (Au, Co, Cu, Ni) forms the nanopillars while the BTO forms the matrix. The difference in growth morphology can be attributed to the difference in the surface energy of the films with the substrate. The surface energy of the metal elements, matrix, and substrate are shown in Table S3. From there, the higher surface energy of the metals as compared to the oxides causes them to undergo Volmer‐Weber growth, which leads to form the pillars. However, the surface energy of the BTO is comparable to that of the substrate STO which causes them to undergo the Frank‐van der Merwe Growth, leading to layered deposition and thus forming the matrix. The pillar diameter of all the films was calculated and summarized in Table S4. The pillar diameters are thin (4.5–7 nm) and are achieved in a single‐step growth. Such fine pillars are hard to achieve by templated growth or nanofabrication. The films in both the temperature systems exhibit an interesting trend that, with the increase in the deposition frequency, the pillar diameter decreases while the pillar density increases. This indicates that the deposition frequency serves as an effective parameter for tuning the nanostructure of the films as previously reported [39]. To further understand the elemental distribution in the nanopillars, atomic resolution STEM images and corresponding EDS maps along with line profiles of two films (600°C, 10 Hz and 750°C, 10 Hz) are shown in Figure 3. Both films reveal a core–shell configuration comprising a Co–Ni magnetic core encapsulated within an Au–Cu optically active shell, establishing a BTO/Au/Cu/Ni–Co interface sequence with Ba–Au and Ni–Cu interfacial bonding. Interestingly, the two different temperature systems show pillars of two different shapes, that is, the lower temperature (600°C) samples have circular pillars, and the higher temperature systems exhibit a faceted pillar structure.

**FIGURE 4:**
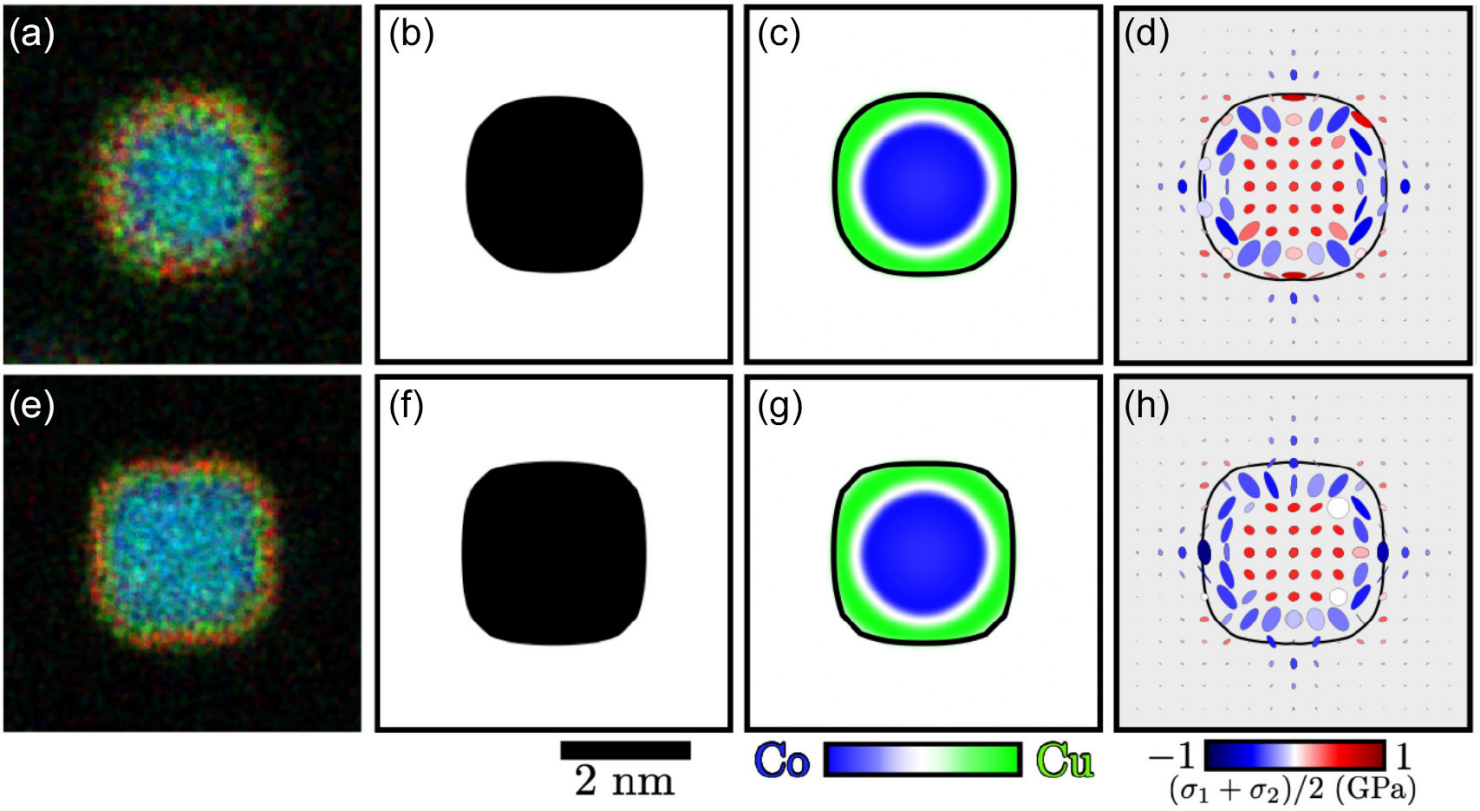
The Co core and Cu shell morphology of a Vertically Aligned Nanopillar embedded in a BaTiO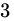 matrix for short times. The top row corresponds to T=873 K (600°C), and the bottom row to T=1023 K (750°C). Insets (a) and (e) correspond to the experimental image of the core–shell morphology of a pillar. Insets (b) and (f) correspond to the simulated equilibrium shape of insets (a) and (e). Insets (c) and (g) correspond to the phase field simulation of the Co and Cu concentration distribution . Insets (d) and (h) correspond to the Lamé ellipses stress distribution as a result of the chemical distribution in (c) and (g). The orientation of the ellipses corresponds to the direction of the maximum principal stress and strain. The maximum shear stress and strain are ±45° away from the principal axes. Despite having similar CoCu distribution for short times, the equilibrium pillar shape influences the resultant chemomechanical stresses.

The equilibrium shape of the pillar is a result of the interplay between surface energies of the pillar and the matrix and the stress along the pillar‐matrix interface, in agreement with Larche and Cahn [40]. Following the work of Finot and Suresh, the interfacial stress state of the pillar is a result of the contributions from the substrate epitaxial strain and the thermal expansion mismatch between the SrTiO_3_ substrate and the BaTiO_3_ matrix, see Figure S18 in the supplemental for details [41]. At T=873 K (600°C), the epitaxial stress dominates the total energy and results in a near circular shape, see Figure 4b. At T=1023 K (750°C), the thermal expansion and the epitaxial strains cancel each other out, resulting on the formation of faceted and elongated pillar, see Figure 4f.

**FIGURE 5:**
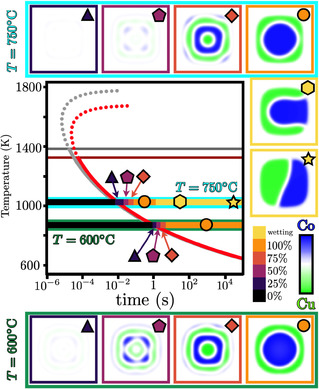
Time, temperature, and phase transformation (TTT) diagram of CoCu Vertically Aligned Nanopillar (VAN) embeded in a BaTiO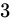 matrix. Specific morphological transformations are shown for T=873 K (600°C), that is, 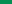, and at T=1023 K (750°C), that is, 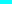. 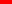 corresponds to the line of initial phase separation stage for stressed VAN, and 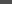 to the initial phase separation stage for stress‐free VAN. Dashed line corresponds to a metastable (super heated) phase separation stage beyond the peritectic temperature. 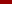 corresponds to the peritectic temperature of the stressed VAN pillar, and 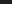 to the stress‐free VAN pillar. 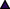 corresponds to the initial stage of the phase separation; 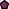 to the acceleration stage of the spinodal characteristic wavelength amplification; 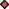 to the final stage of the spinodal characteristic wavelength amplification; 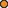 to the formation of the core–shell morphology; 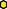 to the wetting phenomenon of the Co core; and 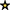 to the equilibrium morphology of the pillar after long times. The TTT diagram of the VAN structure provides a guideline to tune the desired macroscale properties based on temperature and stress state of the VAN composite.

Calculations show that the phase separation kinetics are controlled by the interplay between thermochemical, mechanical, and interfacial driving forces. While the core–shell morphologies are observed for both equilibrium shapes for short times, see Figure 4c,g, the resultant chemomechanical stresses are different as a result of the shape of the pillar. The apparent symmetry of the internal concentration segregation is nonexistent because of the random noise initial condition around c=0.5, and because the amplification factor and the characteristic wavelength is equal in all directions, given the crystallographic anisotropies of both the pillar and the matrix, each displaying four fold symmetry and favoring symmetric concentration segregation. This is most apparent in Figure 4g,h where the Cu‐rich phase diffuses to the diagonal facets, that is, the crystallographically soft direction of the crystal. Combined with the randomness of the noise, this results in the long‐term wetting of the Co‐rich phase as Cu continues to diffuse toward the diagonal direction, see Figure 5.

Using dimensional analysis, the characteristic time for the onset of phase separation is related to the mobility, the Gibbs free energy density, the gradient energy coefficient, and the pillar geometry, in agreement with Martínez [42]. This time, referred herein as the spinodal time, is used to construct the time‐temperature‐transformation diagram, as shown in Figure 5. Phase separation kinetics is classified into five regimes: (1) the critical spinodal regime where compositional fluctuations start to be amplified, 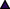; (2) the amplification regime where the fastest growing characteristic wavelength develops, 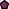; (3) the coarsening regime where segregated compositional domains are established, 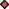; (4) the final regime where the core–shell morphology is stabilized, 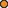; and (5) the wetting regime where the contributions to the interfacial and the mechanical energies result in the Co segregation to the interface.

As one would expect, the driving force for spinodal decomposition increases as the sample cools down; however, the diffusivity and atomic mobility decreases as thermal energy decreases. As a result, an optimum temperature exists where the time for the onset of spinodal decomposition is minimized. Self‐induced chemomechanical stresses are capable of extending the time for phase separation. The peritectic line limits the possibility to reach this limit. Further, the peritectic temperature is also suppressed by the development of composition‐dependent stresses, decreasing it from T=1385 K to T=1326 K. This is because the elastic energy favors the mixing of Co and Cu in order to relieve the internal chemomechanical stresses in the solid. This provides insight into the nanoscale phase separation behavior in complex alloy systems, and the resulting structure–property correlations and their influence on the optical and magnetic responses are systematically analyzed.

**FIGURE 6:**
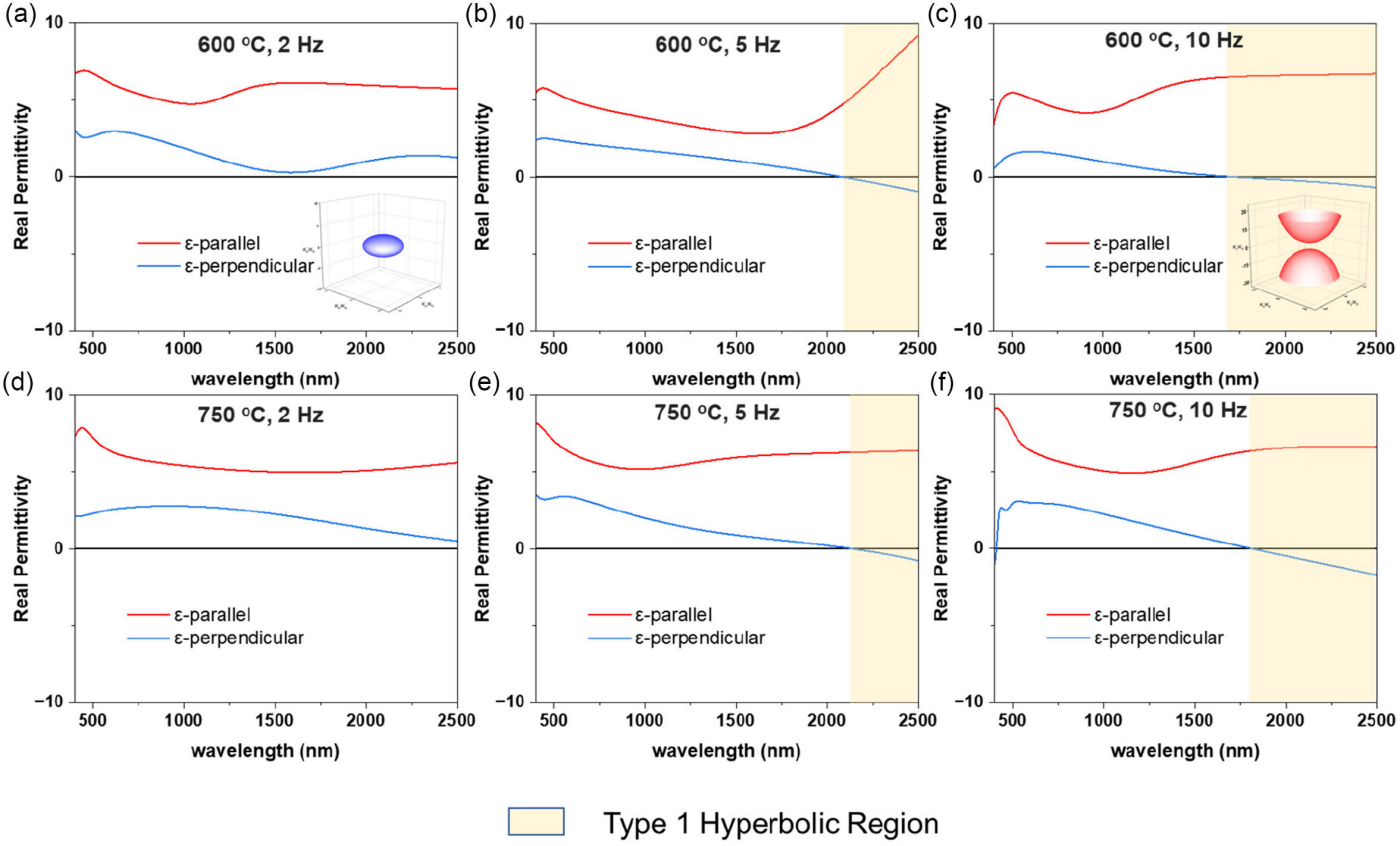
Real part of the complex dielectric function (*ε′*) of the alloy VAN films measured in the OP and in‐plane (IP) directions. The films were deposited at 600°C with laser frequencies of (a) 2 Hz, (b) 5 Hz, and (c) 10 Hz, and at 750°C with frequencies of (d) 2 Hz, (e) 5 Hz, and (f) 10 Hz.

Embedding alloy nanopillars within a dielectric matrix can result in an anisotropic optical response. To evaluate and understand how the change in the microstructure impacts the optical response, angular dependent ellipsometry measurements were conducted from the wavelength of 200–2500 nm on all alloy VAN films using various angles ranging from 30° to 70°. The optical permittivity responses in both directions (*ε*_||_ and *ε*_⊥_) of all the films were obtained by fitting the ellipsometry data through oscillator models for maintaining Kramers‐Kronig consistency as shown in Figure 6. The results show that the *ε*_||_ is positive in all the films, while the *ε*_⊥_ starts to become negative in a particular wavelength, which confirms the hyperbolic nature of the films. In both the temperature systems, we see a blue shift in the eplison‐near‐zero (ENZ) wavelength, with the 2 Hz samples not showing any ENZ wavelength within 2500 nm, the 5 Hz samples showing ENZ wavelengths after 2000 nm and finally the 10 Hz samples exhibiting them before 2000 nm. In metal–oxide VAN structures, there is an anisotropic distribution of the electronic density of states (DOS) in the film as the pillars tend to grow in the OP direction while matrix dominates in the in‐plane (IP) direction. This leads to direction‐dependent permittivity, as metallic VAN pillars with a stronger free‐carrier response, resulting in negative permittivity, have a stronger role in the overall OP permittivity. However, in the IP direction the dielectric matrix, with positive permittivity due to its insulating nature, overshadows the role of the pillars in the IP permittivity due to its layered growth. The blue‐shift of the ENZ wavelength indicates the increasing metallic characteristics of the film, becoming more dominant with rising laser frequency. This is consistent with the PV results shown in Figure 3 where the density of the alloy metal pillars increases with an increase in frequency, thus they are able to increase their OP permittivity contribution. To further examine this, we can utilize the Maxwell‐Garnett theory in the system, where the $\varepsilon_{⊥}=p\varepsilon_{m}+(1-p)\;\varepsilon_{d}\;\mathrm{and}\;\varepsilon_{\left|\right|}=\varepsilon_{d}+\frac{p\varepsilon_{d}(\varepsilon_{m}-\varepsilon_{d})}{\varepsilon_{d}+(1-p)(\varepsilon_{m}-\varepsilon_{d})q_{\mathrm{eff}}}$; where *p* denotes the metal filling ratio of the nanopillars, *ε*_m_ is the metal permittivity, while *ε*_d_ is dielectric permittivity, and finally *q*_eff_ represents effective depolarization factor normal to the nanopillar orientation. As the permittivity of the metal and the dielectric does not change for the films due to only changes in deposition condition, only the filling ratio plays a major role in *ε*_⊥_. Thus, with the rising metal content in the film with the increase in frequency, the filling ratio increases contributing to the negative *ε*_⊥_. The effective complex refractive index comprising the refractive index (n) and extinction coefficient (k) of all the films are plotted in Figure S14. We can better understand the optical anisotropy by visualizing the optical isofrequency surfaces. For electromagnetic wave propagation in a uniaxially anisotropic medium (*ε*_[100]_ = *ε*_[100]_ ≠ *ε*_[100]_), the isofrequency surfaces can be defined by the corresponding dispersion relation $\frac{k_{x}^{2}+k_{y}^{2}}{\varepsilon_{⊥}}+\frac{k_{z}^{2}}{\varepsilon_{∥}}=\frac{\omega^{2}}{c^{2}}=k_{0}^{2}$ . Here, k_*x*_, k_*y*_, and k_z_ represent the wave vector components along the [100,010] and [001] crystallographic directions, while; *ω* is the angular frequency, and c represents the light's speed in vacuum. For comparison, the isofrequency k‐space surfaces at 2300 nm for the films of 2 and 10 Hz at 600°C are presented in the inset of the Figure 6. The geometries changes for the different films with 2 Hz samples having an ellipsoid indicating elliptic dispersion behavior, while the 10 Hz sample exhibits a two‐sheet hyperboloid indicating Type I hyperbolic behavior. The optical anisotropic nature is further evidenced by the reflection intensity measurement, where different reflection intensities were obtained at different angles, plotted in Figure S15. Thus, the alloy VAN films in both the deposition temperature regimes exhibit frequency‐dependent tunable anisotropy, allowing for dynamic control over the transition between elliptic and hyperbolic dispersion, which is crucial for applications in reconfigurable nanophotonics and subwavelength optics [43].

**FIGURE 7:**
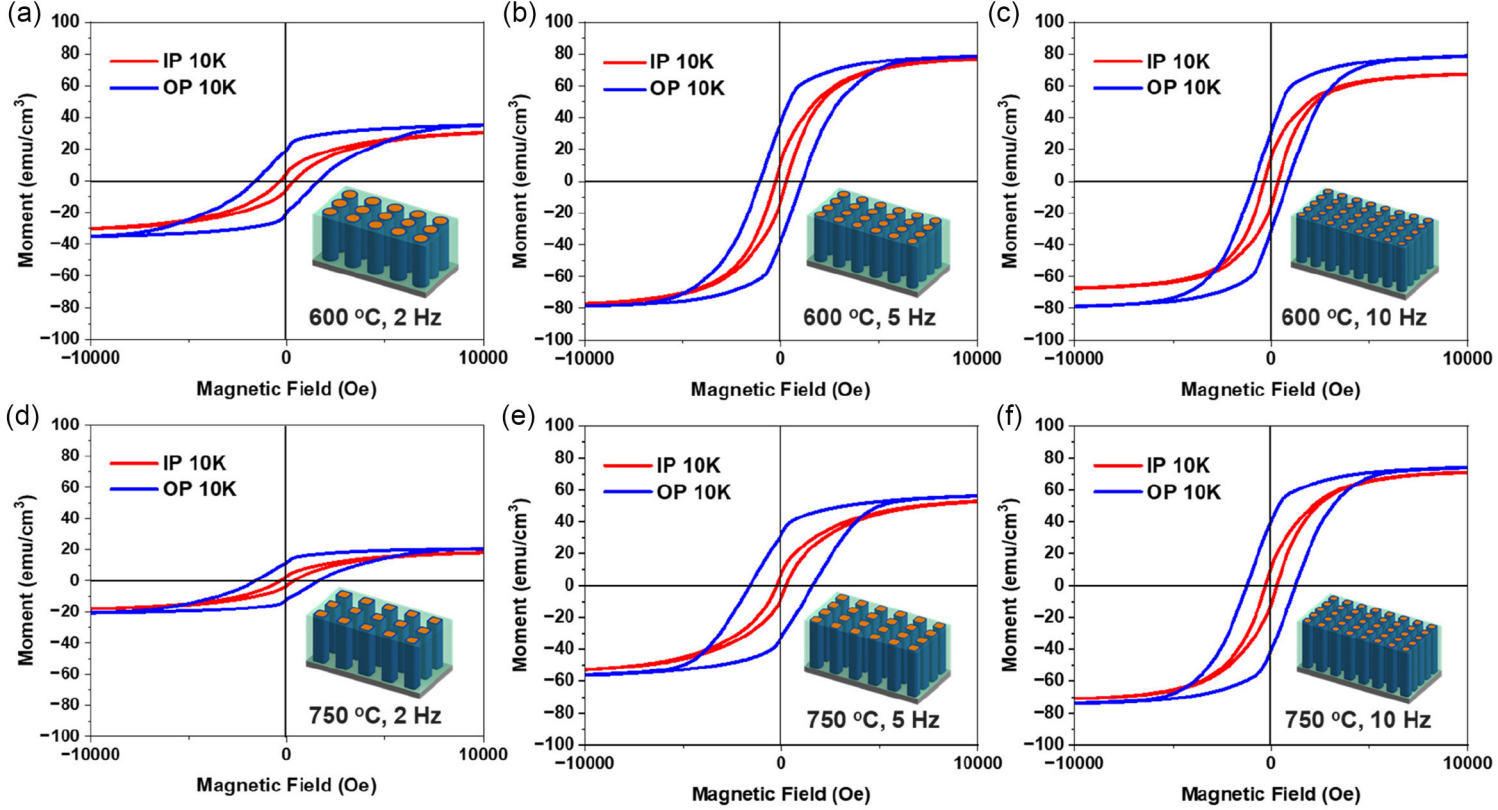
Magnetic hysteresis loops of the Alloy VAN film deposited at 600°C with frequencies of (a) 2 Hz, (b) 5 Hz, and (c) 10 Hz, and at 750°C with frequencies of (d) 2 Hz, (e) 5 Hz, and (f) 10 Hz at 10 K.

To investigate the magnetic potential of the films arising from the ferromagnetic nature of its Co‐Ni core as well as to evaluate the anisotropic nature of the films, magnetic measurements were done with applied field oriented both normal (out‐of‐plane, OP) and parallel (in‐plane, IP) to the surface of the alloy VAN films. The M‐H hysteresis loops of all the deposited samples at 10 K are shown in Figure 7. All the films exhibit magnetic anisotropy as M‐H loops are different for both IP and OP directions, with all the films showing higher coercivity in OP direction. The 2 Hz sample exhibits the highest coercivity in both IP and OP directions for their respective temperature systems, while the 10 Hz sample shows the highest saturation magnetization across both systems. The higher coercivity in 2 Hz sample is because of larger alloy nanopillars, as evidenced by the diameter measurements from PV images. It is also observed that, in both temperature systems, the coercivity in both IP and OP directions generally decreases with decreasing pillar diameter. From previous reports, it is reported that the diameter plays a critical role in coercivity for metal nanowires with a general trend of decreasing coercivity as the diameter increases [44, 45]. This is due to transition from single‐domain to multidomain states in the larger diameter nanowires, which lessens the energy barrier for magnetization reversal. However, our results seem to contrast the previous trends, which could be attributed to the ultrathin size of the pillars (<7 nm) in which we can consider these narrow nanopillars to be magnetic structures with a single domain. Regarding the magnetization reversal process, previous reports have proposed two modes. When the pillar diameter exceeds the critical diameter, the curling mode is followed, while if it is lesser the coherent mode dominates. The critical radius is defined as $R_{c}=\sqrt{kA/\mu_{0}{M_{S}}^{2}}$; here *k* denotes constant, *A* denotes exchange stiffness constant while M_S_ denotes saturation magnetization. Figure 3 suggests that the pillars have a core shell structure with a clear segregation observed between the optically sensitive shell (Au, Cu) and the magnetic core (Co, Ni). If assuming the core consisting of Co‐Ni, the critical radius is calculated to be 4.12 nm, (K = 1.08 for cylinder, A_avg_ of Co‐Ni = 2.27 × 10^−6^ erg/cm, M_S_ = 1070 emu/cm^3^) [46]. Thus, the diameter of the pillars, even including the shell (<7 nm) is less than critical diameter (8.24 nm), in which the coherent rotation mode is followed. Generally, the coercivity remains relatively constant when the diameter is less than the critical diameter. However, in practice, the coercivity often increases with diameter up to critical diameter, after which it decreases due to material defects and thermally activated reversal processes. This is consistent with the trends observed in this case. Also, when we increase the deposition frequency, the pillar density increases and the magnetostatic interaction between pillars increases, also contributing to lower coercivity with higher deposition frequency. The effective magnetic anisotropy of the alloy nanopillars is governed by three factors: shape anisotropy, interpillar dipole interaction, and magnetocrystalline anisotropy. This can be defined as anisotropic field, H_K_ = 2*π*M_S_ – 6.3*π*M_S_r^2^ Ld^–3^ + H_MC_, where L and r are the length and radius of the nanopillars, respectively, and d is the distance between adjacent pillars [47]. Previous studies have shown that Co–Ni nanowire arrays exhibit magnetic anisotropy arising from both shape anisotropy and magneto‐crystalline anisotropy, typically resulting in a weakly defined easy axis along the nanowire (NW) direction [48]. However, in nanowires, the shape anisotropy mostly dominates in comparison with the magneto‐crystalline anisotropy due to the aspect ratio and an alloy nature in our case. From the TEM results, r, L, and d are evaluated to be ≈2.31 nm, ≈41.66 nm and ≈9.45 nm for the 600°C, 10 Hz sample. The H_K_ is calculated to be positive based on the input values. This behavior is consistent with our observations, where the OP coercivity is higher than the IP value. The M‐H hysteresis loops of all the samples at 300 K are shown in Figure S16 where the decrease in coercivity implies the magnetic reversal process is thermally activated. If we correspond each pillar to be one bit, the 10 Hz samples in both the temperature systems will be of great interest due to its higher pillar density, as we can get a maximum theoretical bit density of 7.23 Tbits/inch^2^ for 600°C system and 6.19 Tbits/inch^2^ for the 750°C system. These results demonstrate a promising approach to push the areal density (AD) beyond 5 Tb/sq.in, offering a path toward far higher‐capacity memory devices [49].

**FIGURE 8:**
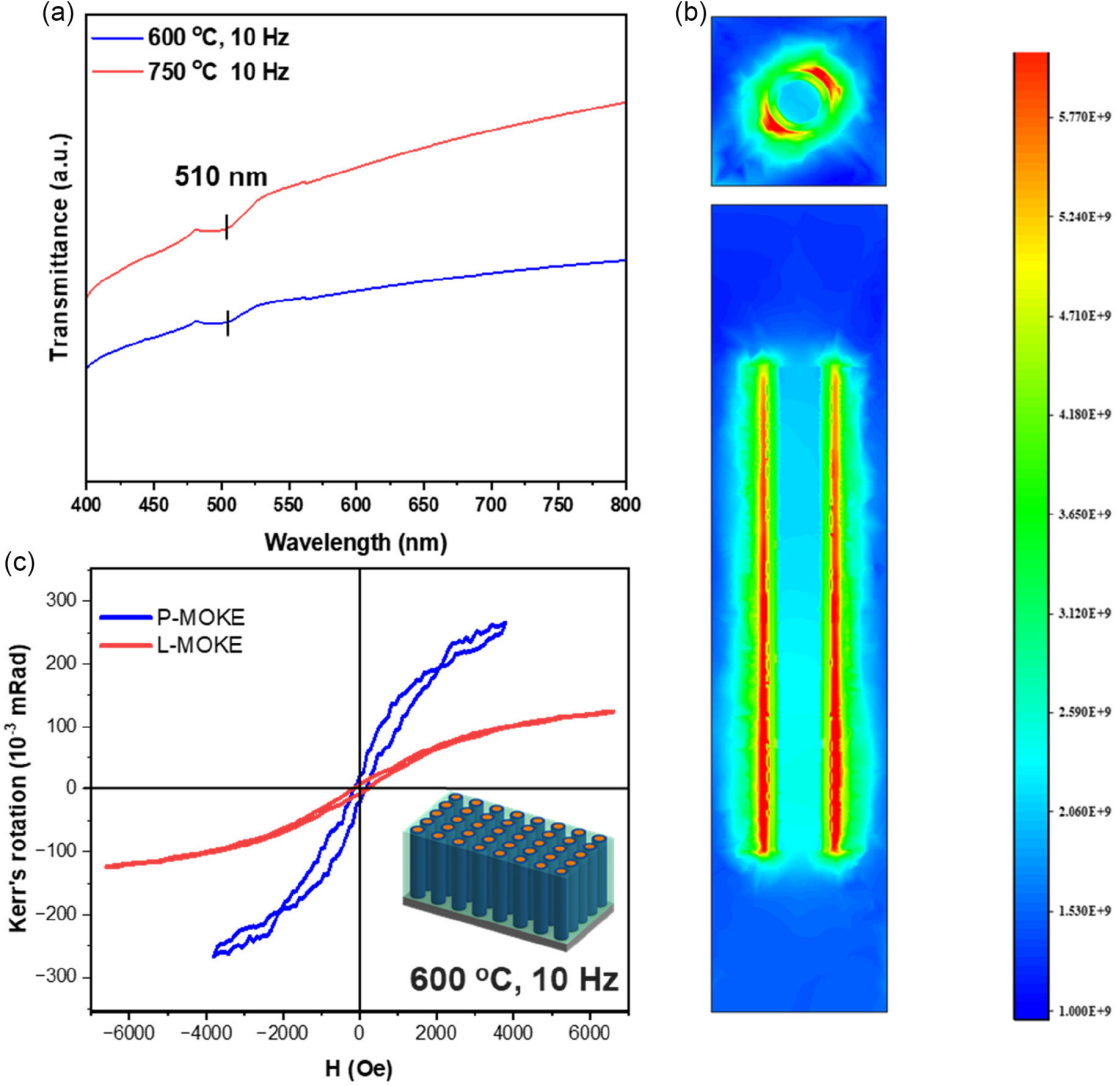
(a) Transmittance spectra of the alloy VAN film deposited at 10 Hz for 600°C and 750°C, (b) Simulated optical field enhancement maps corresponding to incident illumination at 510 nm on an alloy nanopillar, and (c) P‐MOKE and L‐MOKE measured at room temperature.

To further evaluate the multifunctionality of the films, transmittance measurements on 2 films were carried out, which confirm the plasmonic potential of the film as shown in Figure 8a. The spectra shows that both the temperature systems have a SPR at the wavelength of ~510 nm. This could be attributed to the outermost Au shell as validated by the COMSOL simulations in Figure 8b which could allow films to find potential applications in sensors and nanophotonics. The experimental transmittance data also matches with the simulated transmittance spectra obtained from COMSOL model, as shown in Figure S17. Due to the presence of both optical and magnetic potential, the potential coupling was investigated by doing magneto‐optic Kerr effect (MOKE) measurement on a film using two configurations, namely, Polar‐MOKE (P‐MOKE) and Longitudinal‐MOKE (L‐MOKE). P‐MOKE measurement was performed using a perpendicular magnetic field with normally incident laser, whereas L‐MOKE was done with in‐plane magnetic field with the laser incident at an angle of 30°. Although the sample show MOKE response in both configurations, the response in the polar side is higher than the longitudinal side. This could be attributed to the higher coupling at vertical interface between the matrix and alloy pillars in OP direction in comparison with the IP direction.

Our work is a significant advancement in the study of complex alloy thin films by demonstrating a strong correlation between material structure and properties through the morphological tuning of alloy VAN systems. In the alloy VAN system, we achieve a multifunctional architecture with various anisotropic attributes through optical, magnetic, MOKE, and plasmonic response in a single platform. This is achieved using a one‐step PLD process to grow core–shell alloy nanopillars within an oxide matrix. In the prior Cu‐Co metal alloy VAN work [27], different nanopillar morphologies were observed and attributed to the possible alloy composition variation in different pillar types. Interestingly, the additional elements introduced led to more uniform core–shell morphology despite various temperatures and frequency explored. This multielement design gives much more freedom in the selection of pillar materials, morphology tuning and multifield coupling to conventional and magneto‐plasmonics that largely focuses on multilayers or nanoparticles. The demonstrated synthesis‐to‐property correlation (e.g., pillar diameter and pillar density tunability to tailored hyperbolic regime and magnetic anisotropy in this work) provides a practical framework for optimizing performance toward specific functions. Importantly, the demonstrated property allows for multiple real‐world applications; for example, optical anisotropy and hyperbolic behavior are relevant for nanophotonic and metasurface components, and the coexistence of plasmonic and magneto‐optical responses enables magneto‐plasmonic active optics and sensing. The strong OP magnetic anisotropy further supports new spintronic and nanomagnetic memory concepts that benefit from strong perpendicular anisotropy. The phase field modeling work on alloy pillar growth and morphology evolution provides valuable insights into the interplays between the surface energy, epitaxial strain energy, and thermal expansion strain energy in the final morphology. The study demonstrates a new time‐temperature‐transformation diagram for the alloy pillar formation and morphology which can be used to guide the fabrication process for versatile pillar designs. Future efforts could include exploring the nanostructure growth in diverse gas environments, incorporating other immiscible yet lattice‐matched oxides, integrating the films with other substrates such as Si, mica, and transparent electrodes, and conducting broader studies that systematically vary elemental combinations and compositional ratios. These alloy VAN strategies could functionalize a single, scalable platform for broad applications in electronics, optoelectronics, and flexible devices.

## 3 Conclusion

In this work, Co‐Ni‐Au‐Cu 4‐element alloy VAN films were deposited under different deposition conditions. By tuning the deposition parameters, we examined the morphology tuning and the influence of the microstructure on the property of the film. Atomic‐scale STEM characterization confirmed a core–shell structure in both temperature systems, with a ferromagnetic Co‐Ni core and an optically active Au‐Cu shell. The microstructural analyses reveal that the higher deposition frequencies result in smaller pillar diameter with increased pillar density, which influence both optical and magnetic responses. Thermodynamically, the pillar morphology is a result of the interplay between the pillar‐matrix interfacial energy and the elastic energy, where a stress‐dominated system produces circular pillar, and interfacial energy‐dominated system results in faceted pillar. Additionally, a phase field and dimensional analysis on the kinetics of phase saparation of  the metallic pillar results in the construction of a time‐temperature‐transformation diagram that allows the prediction of the pillar processing time. Results also show that the self‐induced chemomechanical stresses not only extend the time for phase separation but also suppress the peritectic temperature. Ellipsometry measurements show that the films have anisotropic behavior with tunable hyperbolic region near infrared. Magnetic measurements reveal the films have a stronger OP anisotropy and tunable magnetic behavior as coercivity decreases by increasing the deposition frequency. Additionally, plasmonic resonance and MOKE response were also observed. All these findings demonstrate the tunability and multifunctionality of alloy VAN platforms. This capability for engineering nanoscale phase separation to achieve the optimum microstructure and strongly coupled properties creates new avenues for integrating these materials into the next generational device architectures.

## 4 Experimental Details

### 4.1 Thin Film Deposition

The alloy VAN films were deposited from a mixed composite target with a molar ratio of BTO:Au:Co:Cu:Ni = 0.5:0.125:0.125:0.125:0.125 prepared by the solid‐state mixing, which was followed by spark plasma sintering (Thermal Technology 10–3 SPS) done at a temperature of 200°C and pressure of 30 MPa without holding time. The films were deposited on STO (001) substrates with a size of 10 × 5 mm by PLD having a KrF excimer laser (Lambda Physik Compex Pro 205, *λ* = 248 nm). The deposition was started after evacuating the chamber till pressure was below 2 × 10^−6^ torr and no background gas was added. The substrates were kept at temperatures of 600°C and 750°C with the laser frequencies of 2, 5 and 10 Hz, yielding a total of six samples. The laser energy was set at ~2 J/cm^−2^. The work involved deposition of multiple test samples to carry out parameter and growth optimization, after which the six samples reported here were selected. The samples were cooled naturally in vacuum in the chamber after deposition.

### 4.2 Structural Characterization

*θ*–2*θ* and RSM scans were done by XRD (Panalytical Empyrean X‐ray diffractometer). STEM and EDX characterizations were conducted by FEI TALOS F200X system, while high resolution EDX characterizations were conducted by FEI Titan G2 80–200 ChemiSTEM at 200 kV. The TEM samples were prepared manually by thinning them with sandpaper then using dimpling and finally ion milling (Gatan precision ion polishing system).

### 4.3 Property Characterization and Simulation

Permittivity measurements were obtained by ellipsometer (Woollam RC2). Two parameters (*ψ* and Δ) having a relation *r*_p_/*r*_s_ = tan(*ψ*)e^(iΔ)^ (reflection coefficients of p‐polarization and s‐polarization lights) were measured at various incident angles (35° to 75° with a step of 10°). Then, the refractive index and the permittivity was extracted by data fitting in CompleteEASE software. Magnetic hysteresis measurements were completed with an MPMS Model 3 (Quantum Design) with EverCool SQUID magnetometer in the user facility of the Birck Nanotechnology Center at Purdue University, see birck.research.purdue.edu. The transmittance spectra were obtained by spectrophotometer (PerkinElmer Lambda 1050). The MOKE measurements were done using a home‐built system designed for both polar and longitudinal modes, with a 632.8 nm.

Optical simulation was done using the COMSOL Multiphysics Wave Optics Module with the electromagnetic waves, frequency domain (ewfd) interface. The optical constants of metals and matrix were obtained directly from the software database, and the simulated model was made based on plan‐view and cross‐sectional STEM images. A normal‐incidence depolarized electromagnetic field was applied through two ports placed at the top and bottom of the model. The phase‐field modeling information is provided in the Supporting Information.

## Supporting Information

Additional supporting information can be found online in the Supporting Information section. **Supporting Fig. S1:** Cross section STEM image of the 600°C, 2 Hz alloy sample with elemental mapping of the nanopillars. **Supporting Fig. S2:** Cross section STEM image of the 600°C, 5 Hz alloy sample with elemental mapping of the nanopillars. **Supporting Fig. S3:** Cross section STEM image of the 600°C, 10 Hz alloy sample with elemental mapping of the nanopillars. **Supporting Fig. S4:** Plan‐View STEM image of the 600°C, 2 Hz alloy sample with elemental mapping of the nanopillars. **Supporting Fig. S5:** Plan‐View STEM image of the 600°C, 5 Hz alloy sample with elemental mapping of the nanopillars. **Supporting Fig. S6:** Plan‐View STEM image of the 600°C, 10 Hz alloy sample with elemental mapping of the nanopillars. **Supporting Fig. S7** Cross‐section EDS profile of all the alloy VAN films deposited at 750°C with the schematics shown in the inset having deposition frequencies of (a) 2 Hz, (b) 5 Hz, and (c) 10 Hz. The corresponding plan‐view images of the same temperature are shown in (d) 2 Hz, (e) 5 Hz, and (f) 10 Hz. **Supporting Fig. S8:** Cross section STEM image of the 750°C, 2 Hz alloy sample with elemental mapping of the nanopillars. **Supporting Fig. S9:** Cross section STEM image of the 750°C, 5 Hz alloy sample with elemental mapping of the nanopillars. **Supporting Fig. S10:** Cross section STEM image of the 750°C, 10 Hz alloy sample with elemental mapping of the nanopillars. **Supporting Fig. S11:** Plan‐View STEM image of the 750°C, 2 Hz alloy sample with elemental mapping of the nanopillars. **Supporting Fig. S12:** Plan‐View STEM image of the 750°C, 5 Hz alloy sample with elemental mapping of the nanopillars. **Supporting Fig. S13:** Plan‐View STEM image of the 750°C, 10 Hz alloy sample with elemental mapping of the nanopillars. **Supporting Fig. S14:** Refractive index (n) and extinction coefficient (k) of all the alloy VAN films. The films were deposited at 600°C with laser frequencies of (a) 2 Hz, (b) 5 Hz, and (c) 10 Hz, and at 750°C with frequencies of (d) 2 Hz, (e) 5 Hz, and (f) 10 Hz. **Supporting Fig. S15:** Reflection Intensities at different angles of all the alloy VAN films. The films were deposited at 600°C with laser frequencies of (a) 2 Hz, (b) 5 Hz, and (c) 10 Hz, and at 750°C with frequencies of (d) 2 Hz, (e) 5 Hz, and (f) 10 Hz. **Supporting Fig. S16:** Magnetic hysteresis loops of the Alloy VAN film deposited at 600°C with frequencies of (a) 2 Hz, (b) 5 Hz, and (c) 10 Hz, and at 750°C with frequencies of (d) 2 Hz, (e) 5 Hz, and (f) 10 Hz at 300 K. **Supporting Fig. S17:** Diameter histograms of nanopillars measured from plan‐view TEM for alloy VAN films. **Supporting Fig. S17:** Simulated Transmittance spectra from COMSOL model. **Supporting Fig.** **S1** **8** **:** The stress profile of a bilayer due to the epitaxial and thermal expansion coefficient mismatch between the substrate and the pillar‐matrix film. 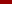 corresponds to the stress in the bilayer at *T*=873 K, and 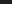 at *T*=1023 K. The interplay between the thermal and epitaxial stresses results in lower compression stress state inside the pillar‐matrix film, which in turn determines the equilibrium shape of the pillar. **Supporting Fig.** **S1** **9** **:** (a) q‐2q XRD scans of pure BTO film grown and cooled in Vacuum (750°C, 10 Hz) on STO substrate*.* **Supporting Table S1:** List of parameters used in the phase field model. **Supporting Table S2:** List of parameters used in the bilayer stresses computation. **Supporting Table S3:** Surface energies of all the materials in the film. **Supporting Table S4:** Pillar Diameters of all the films calculated based on plan‐view STEM images. **Supporting Table S5:** BTO lattice parameters calculated. **Supporting Table S5:** FWHMx values of the films.

## Author Contributions

**Abhijeet Choudhury**: conceptualization, methodology, investigation, formal analysis, validation, visualization, writing – original draft. **Abhijeet Choudhury** and **James P. Barnard**: nanostructure characterization, formal analysis. **Jeremy Gan, Jialong Huang,** and **Natalia Garcia Godinez**: optical property characterization. **Ping Lu, Benson Kunhung Tsai**: TEM images acquisition and analysis. **Lizabeth Quigley, Bharat Giri,** and **Xiaoshan Xu**: magnetic property characterization. **D. Hermawan, A. Sanjuan, César Martínez‐Sánchez, Zedong Hu** and **R. Edwin García**: simulation. **Haiyan Wang**: conceptualization, methodology, writing – review & editing, project administration, funding acquisition, supervision, resources.

## Funding

This study was supported by DMREF (2323752), Office of Naval Research (N00014‐22‐1‐2160).

## Conflicts of Interest

The authors declare no conflicts of interest.

## Supporting information

Supplementary Material

## Data Availability

The data that support the findings of this study are available in the supplementary material of this article.
