# Supplementary material for: Tunable Core–Shell Metal Alloy Pillar Design in Vertically Aligned Nanostructures Toward Multifunctionality
Source: Small Sci. 2026 Mar 19;6(3):e202500622. doi: 10.1002/smsc.202500622 (PMC13098058; doi:10.1002/smsc.202500622)
Supplement: Supplementary file 1 — Supplementary Material [file SMSC-6-e202500622-s001.pdf]

Supporting Information

Tunable Core-shell Metal Alloy Pillar Design in Vertically Aligned Nanostructures (VANs) towards Multifunctionality

Abhijeet Choudhury[1], Benson Kunhung Tsai[1], Ping Lu[2, 3], D. Hermawan[1], Lizabeth Quigley[1], Jialong Huang[1], Zedong Hu[4], Jeremy Gan[1], Natalia Garcia Godinez[1], James P. Barnard[1], Bharat Giri[5], A. Sanjuan[1], C. Martínez[6], Xiaoshan Xu[5], R. Edwin García[1], Haiyan Wang[1, 4]

[1]*School of Materials Engineering, Purdue University, West Lafayette, Indiana 47907, United States*
[2] *Sandia National Laboratories, Albuquerque, NM 87185, USA*
[3] *Center for Integrated Nanotechnologies, Sandia National Laboratories, Albuquerque, New Mexico 87185, USA*
[4] *School of Electrical and Computer Engineering, Purdue University, West Lafayette, Indiana 47907, United States*
[5] *Department of Physics and Astronomy, University of Nebraska, Lincoln, NE 68588, USA*
[6] *Department of Physics, Universidad Nacional Autónoma de México, Mexico City 04510, Mexico*

*Corresponding Author: hwang00@purdue.edu

Theoretical Framework 1

The equilibrium shape of a stressed nanopillar embedded in a matrix is mathematically defined as:

$$W_\sigma = \min\{\vec{x}: W_\mathrm{M}(\vec{x}) + (\overleftrightarrow{\sigma}_{int}\ \delta \cdot \hat{n}) + (\overleftrightarrow{\sigma}_{int}\ \delta \cdot \hat{t})\} \tag{1}$$

where W_σ is the equilibrium shape of a stressed VAN, W_M is the equilibrium shape of an unstressed pillar-matrix interface, $\overleftrightarrow{\sigma}_{int}$ is the interfacial stress tensor, δ is the interfacial width, $\hat{n}$ is the unit interface normal vector, and $\hat{t}$ is the unit interface tangent vector. The equilibrium shape that results from combining the pillar and matrix crystal shapes, W_M, is $W_\mathrm{M} = \min\{\vec{x}: \vec{x}_p + \vec{x}_m$ for every $\vec{x}_p$ in W_p and $\vec{x}_m$ in $W_m\}$, , where W_p is the equilibrium crystal shape of the pillar in vacuum, and W_m is the equilibrium crystal shape of the matrix. This is mathematically defined as $W_i = \min\{\vec{x}: \vec{x} \cdot \hat{n} \leq \gamma_i(\hat{n})$ for all $\hat{n}\}$, , where $\gamma_i(\hat{n})$ is the surface energy as a function of the surface normal, $\hat{n}$.

Define a metallic alloy nanopillar phase embedded in a ceramic in terms of order parameter ϕ, so that $\phi = 1$ is the metallic phase and $\phi = 0$ is the ceramic phase. The local composition is $c_\mathrm{Cu} + c_\mathrm{Co} = 1$. For simplicity, $c_\mathrm{Cu} = c$. The total Gibbs free energy of an isothermal, two-component system, $G_{total}[c, \vec{u}, \phi, T]$, is given by temperature, T, elastic stress, $\overleftrightarrow{\sigma}$, and elastic strain, $\overleftrightarrow{\varepsilon}_e$, defined by the displacement vector, $\vec{u}$:

$$G_{total}[c, \vec{u}; \phi, T] = \int_V \left[g(c; \phi, T) + \frac{1}{2}\kappa_\phi (\nabla\phi)^2 + \frac{1}{2}\kappa_c (\nabla c)^2 + \frac{1}{2}\overleftrightarrow{\sigma} \cdot \overleftrightarrow{\varepsilon}_e \right] dV \tag{2}$$

where $g(c; \phi, T) = p(\phi) g_\mathrm{VAN} + \big(1 - p(\phi)\big) g_\mathrm{BTO} + W\phi^2(1-\phi)^2$ is the volumetric Gibbs free energy density, $\frac{1}{2}\kappa_\phi(\nabla\phi)^2$ is the interfacial energy penalty for the pillar-matrix interface, $\frac{1}{2}\kappa_c(\nabla c)^2$ is the gradient energy penalty for Cu-Co phase boundary, and $\frac{1}{2}\overleftrightarrow{\sigma} \cdot \overleftrightarrow{\varepsilon}_e$ is the mechanical energy density. Also, $g_\mathrm{VAN} = \Delta g_\mathrm{Cu} c + \Delta g_\mathrm{Co}(1-c) + RT\big(c\log(c) + (1-c)\log(1-c)\big) + c(1-c)(L_0 + (2c-1)L_1)$, a Redlich-Kister free energy description , where Δg_i is the free energy of formation for ith component, R is the universal gas constant, and L_n for $n = \{0,1\}$ is the interaction parameters. The elastic stress is described by Hooke's law, $\sigma_{ij} = C_{ijkl}(\phi)\varepsilon_{e,kl}$, where $C_{ijkl}(\phi) = C^\mathrm{p}_{ijkl} p(\phi) + C^\mathrm{m}_{ijkl}\big(1 - p(\phi)\big)$ is the phase-dependent stiffness tensor, $\varepsilon_{e,kl} = \varepsilon^T_{kl} - p(\phi)\beta_{kl}(c - c_\circ)$ is the elastic strain, $\varepsilon^T_{kl} = \frac{1}{2}\left(\frac{\partial u_i}{\partial x_j} + \frac{\partial u_j}{\partial x_i}\right)$ is the geometrical strain in the small deformation limit, β_{kl} is the chemical expansion coefficient inside the pillar, and $c_\circ$ is the stress-free concentration.

Equilibrium is defined in terms of the variational derivatives of Equation (2):

$$\begin{aligned} \frac{\delta G}{\delta c} &= \mu = \frac{\partial g}{\partial c} - \nabla \cdot \kappa_c \nabla c - \sigma_{ij} \cdot p(\phi)\beta_{ij} \\ \frac{\delta G}{\delta \vec{u}} &= \nabla \cdot \overleftrightarrow{\sigma} = \vec{0} \end{aligned} \tag{3}$$

The first row corresponds to the chemomechanical potential. The second row corresponds to the mechanical equilibrium equation.

Away from equilibrium, the kinetic equation for Cu distribution is defined as:

$$\frac{\partial c}{\partial t} = \nabla \cdot M_c \left[\frac{\partial^2 g}{\partial c^2} \nabla c + \frac{\partial^2 g}{\partial c\, \partial \phi} \nabla \phi - \kappa_c \nabla^3 c - \nabla\left(\overleftrightarrow{\sigma} \cdot p(\phi) \overleftrightarrow{\beta} \right) \right] \quad (4)$$

where $M_c = \left(p(\phi)D_\mathrm{p} + \left(1 - p(\phi)\right)D_\mathrm{m}\right)c(1-c)V_m/RT$ is the mobility, D_p is the diffusivity of metal in the pillar, D_p is the diffusivity of metal in the matrix, and V_m is the molar volume.

Numerical Implementation

Equation (1) was numerically implemented in the convex hull module of SciPy 1.12.1 and NumPy 1.26.4 . Each equilibrium shape calculation used 54 MB of RAM and took on the order of five seconds of wall time. A total of 720 interfacial normals were used to construct the equilibrium shape. Used parameters are summarized in Table S1.

The thermodynamic parameters of the volumetric Gibbs free energy density was optimized with machine learning formulation developed by Lund, *et al.* , and experimental data reported in the scientific literature by Kubišta and Vřešťál . The gradient energy coefficient, κ_c, was obtained from the experimental observation of the pillar from previous work, , by using the experimental characteristic wavelength $\lambda \approx 3.7$ nm. The Gibbs free energy density in the matrix, $g_\mathrm{m} = \Delta g_\mathrm{i}^\mathrm{m} c + \Delta g_\mathrm{j}^\mathrm{BTO}(1-c) + RT\left(c\log(c) + (1-c)\log(1-c)\right)$, is set to be thermodynamically unfavorable for solute to diffuse into the matrix.

Equation (4) was solved in FiPy version 3.4.4 , a finite volume partial differential equation solver library in python, and the mechanical equilibrium equation was solved in OOF2 version 2.3.3 , a finite element C++/python library. The physical couplings between mechanical and thermochemical fields were integrated by transferring the order parameter, ϕ, and the concentration, c, from FiPy to OOF2 to compute the phase-dependent stiffness tensor and the chemical expansion inelastic strain, solving the mechanical equilibrium equation, and transferring back the displacement field from OOF2 to FiPy. Scipy interpolation module was used to interpolate the fields between FiPy and OOF2 meshes. A mesh of 20×20 nm simulated region was discretized into 600×600 linear quadrilateral elements in FiPy and 23194 linear triangular elements in OOF2. Simulations were carried out on a 2.7 GHz, 64-cores, Ubuntu 22.04 workstation with 1TB of RAM. The relative tolerance for convergence of the iterative solver is set to 1×10^{-9}. The total wall time for each computation ranges between 8 to 12 hours, and took approximately 8GB of RAM.

Table S1: List of parameters used in the phase field model

Parameter	Value	Units	Ref.
C_{11}^m	275.1	GPa	
C_{12}^m	178.9	GPa	
C_{33}^m	164.8	GPa	

Parameter	Value	Units	Ref.
C_{44}^{m}	54.4	GPa	
C_{66}^{m}	151.6	GPa	
C_{11}^{p}	180	GPa	
C_{12}^{p}	127	GPa	
C_{44}^{p}	78	GPa	
D_m	$D_{\text{VAN}} \times 10^{-6}$	m^2/s	-
D_p	$8.5 \times 10^{-8} e^{(-258000/RT)}$	m^2/s	
L_0	34589 - 2.81 T	J/mol	-
L_1	1974 - 0.922 T	J/mol	-
T	873, 1023	K	-
$T_{m,\text{Co}}$	1768	K	
$T_{m,\text{Cu}}$	1358	K	
V_m	6.9811×10^{-6}	m^3/mol	-
β_{ij}	0.0279	-	
Δg_{Co}	$-18941(1 - T/T_{m,\text{Co}})$	J/mol	-
Δg_{Cu}	$-15949(1 - T/T_{m,\text{Cu}})$	J/mol	-
$\Delta g_{\text{Co}}^{\text{BTO}}$	40000	J/mol	-
$\Delta g_{\text{Cu}}^{\text{BTO}}$	40000	J/mol	-
γ_{100}^{Cu}	1.448	J/m^2	
γ_{110}^{Cu}	1.434	J/m^2	
γ_{111}^{Cu}	1.477	J/m^2	
γ_{100}^{m}	1.1	J/m^2	
γ_{110}^{m}	$1.45\gamma_{100}^{m}$	J/m^2	
γ_{111}^{m}	$1.45\gamma_{100}^{m}$	J/m^2	
κ_c	1.1×10^{-10}	J/m	

Theoretical Framework 2

Consider a bilayer of dissimilar solids thin films subjected to internal inelastic strain, , $\vec{\varepsilon}_{inelastic}$. In agreement with Finot and Suresh [19], the geometrical strain, $\varepsilon_{xx}^{g} = \varepsilon_{yy}^{g} = \varepsilon_{g} = \varepsilon_{\circ} + \kappa_{g} h$, is a function of the volumetric deformation, $\varepsilon_{\circ}$, bending strain, κh, the film curvature, κ_{g}, and the film thickness, h. The equi-biaxial stress in each i-th layer is described as $\sigma_{xx,i} = \sigma_{yy,i} = \sigma_{i} = \frac{E_i}{1-\nu_i}\left(\varepsilon_g - \alpha_i \Delta T - \Delta\varepsilon_{epitaxial,i}\right)$, where α is the thermal expansion coefficient, $\Delta\varepsilon_{epitaxial} = \frac{a_i - a_{substrate}}{a_{substrate}}$ is the epitaxial strain, and a is the lattice parameter. The stresses are a function of the film thickness only in regions far away from the free edges. The net forces and moments equilibrium conditions give:

$$\begin{gathered} \int_{h_1}^{h_2} \sigma_2(h)\, dh + \int_{0}^{h_1} \sigma_1(h)\, dh = 0 \\ \int_{h_1}^{h_2} \sigma_2(h)\, h\, dh + \int_{0}^{h_1} \sigma_1(h)\, h\, dh = 0 \end{gathered} \tag{5}$$

By solving Equation (5) for $\varepsilon_{\circ}$ and κ_{g}, the stresses across the bilayer thickness can be computed, which are shown in Figure S18. The list of parameters used to compute the bilayer stresses were summarized in Table S2. The Young's modulus and Poisson's ratio of anisotropic system was approximated by solving $0 = (C_{12} + 2C_{44})\nu + 2(C_{12} + C_{44})\nu^2 - C_{12}$ for ν and $E = 2C_{44}(1+\nu)$. The properties of the pillar were computed by averaging the values from each component. The properties of pillar-matrix layer were computed by using a rule of mixture, where the pillar phase fraction was 0.17 based on the plan view of the microstructural image.

Table S2: *List of parameters used in the bilayer stresses computation*

Parameter	Value	Units	Ref.
a_{Au}	4.08×10^{-10}	m	[18]
a_{BTO}	4.02×10^{-10}	m	[16]
a_{Co}	3.544×10^{-10}	m	[6]
a_{Cu}	3.615×10^{-10}	m	[19]
a_{Ni}	3.48×10^{-10}	m	[19]
a_{STO}	3.9×10^{-10}	m	[20]
C_{11}^{Au}	192	GPa	[21]
C_{12}^{Au}	163	GPa	[21]
C_{44}^{Au}	42	GPa	[21]
C_{11}^{BTO}	275.1	GPa	
C_{12}^{BTO}	178.9	GPa	
C_{33}^{BTO}	164.8	GPa	

Parameter	Value	Units	Ref.
a_{Au}	4.08×10^{-10}	m	[18]
C_{44}^{BTO}	54.4	GPa	
C_{66}^{BTO}	151.6	GPa	
C_{11}^{Co}	255	GPa	[22]
C_{12}^{Co}	160	GPa	[22]
C_{44}^{Co}	92	GPa	[22]
C_{11}^{Cu}	180	GPa	[23]
C_{12}^{Cu}	127	GPa	[23]
C_{44}^{Cu}	78	GPa	[23]
C_{11}^{Ni}	250	GPa	[24]
C_{12}^{Ni}	151	GPa	[24]
C_{44}^{Ni}	122	GPa	[24]
C_{11}^{STO}	283	GPa	[20]
C_{12}^{STO}	97.5	GPa	[20]
C_{44}^{STO}	117	GPa	[20]
α_{BTO}	1.1×10^{-5}	K^{-1}	[25]
α_{Au}	1.7×10^{-5}	K^{-1}	[26]
α_{Co}	1.4×10^{-5}	K^{-1}	[27]
α_{Cu}	2×10^{-5}	K^{-1}	[28]
α_{Ni}	2.2×10^{-5}	K^{-1}	[29]
α_{STO}	3.2×10^{-5}	K^{-1}	[30]
T	873, 1023	K	-

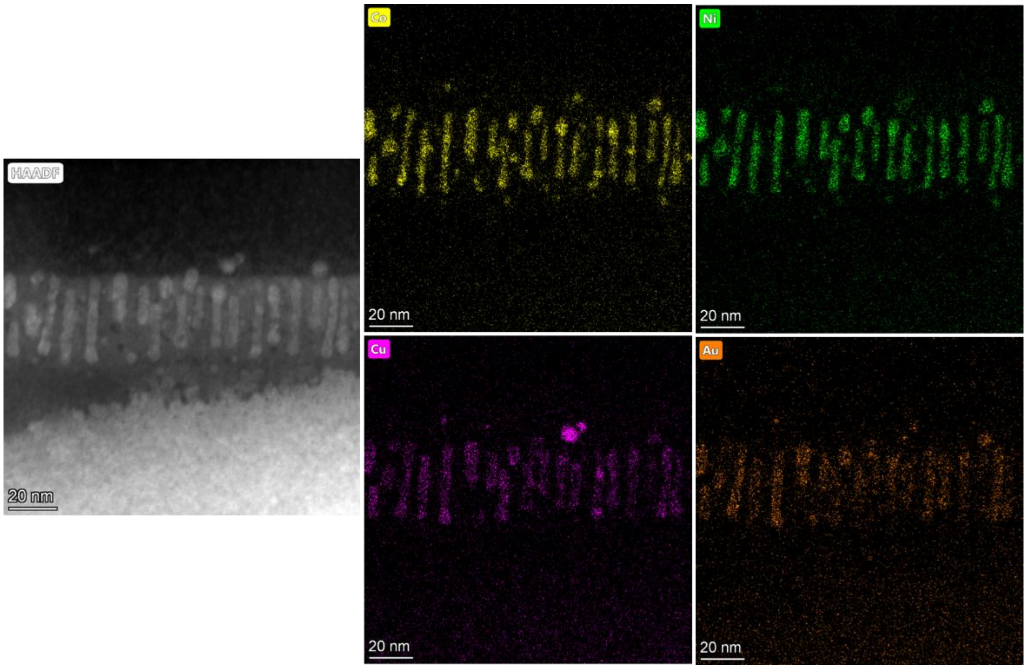

Figure S1: Cross section STEM image of the 600 °C, 2 Hz alloy sample with elemental mapping of the nanopillars

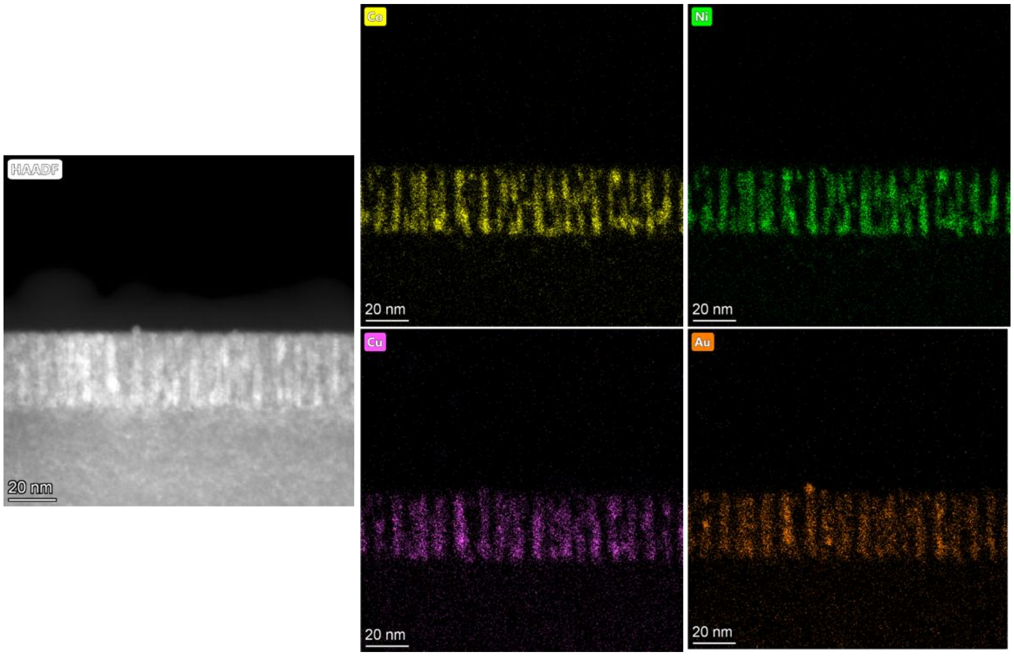

Figure S2: Cross section STEM image of the 600 °C, 5 Hz alloy sample with elemental mapping of the nanopillars

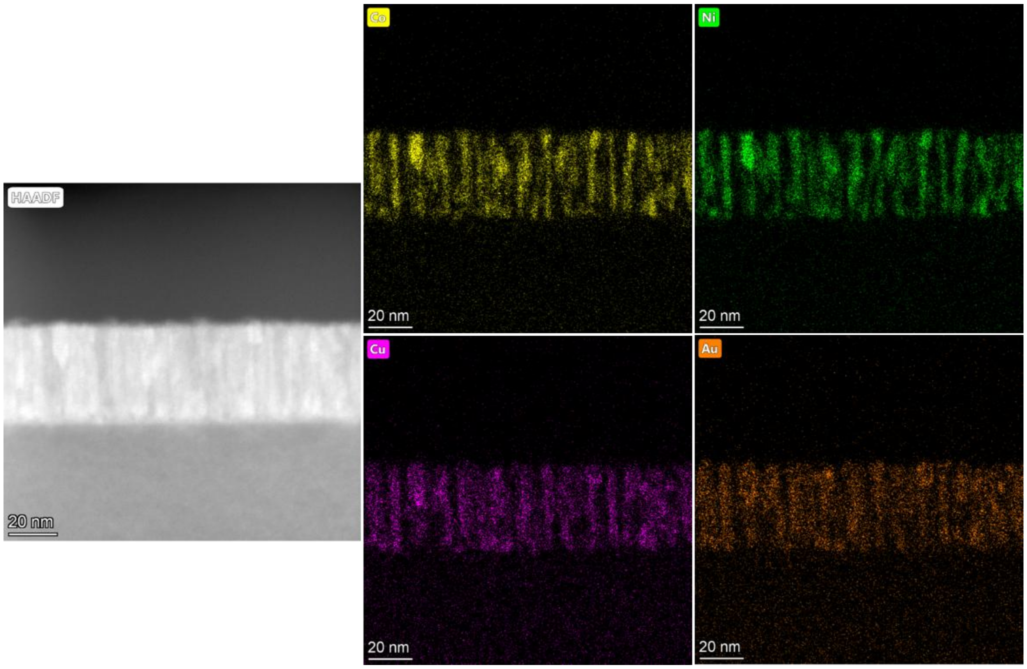

Figure S3: Cross section STEM image of the 600 ^{o}C, 10 Hz alloy sample with elemental mapping of the nanopillars

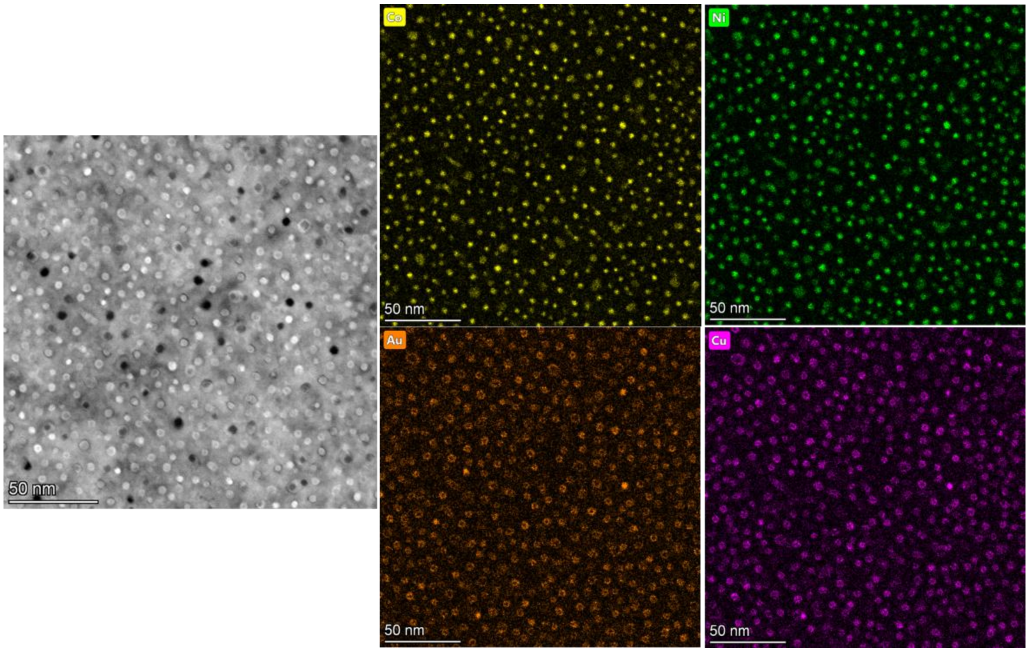

Figure S4: Plan View STEM image of the 600 ^{o}C, 2 Hz alloy sample with elemental mapping of the nanopillars

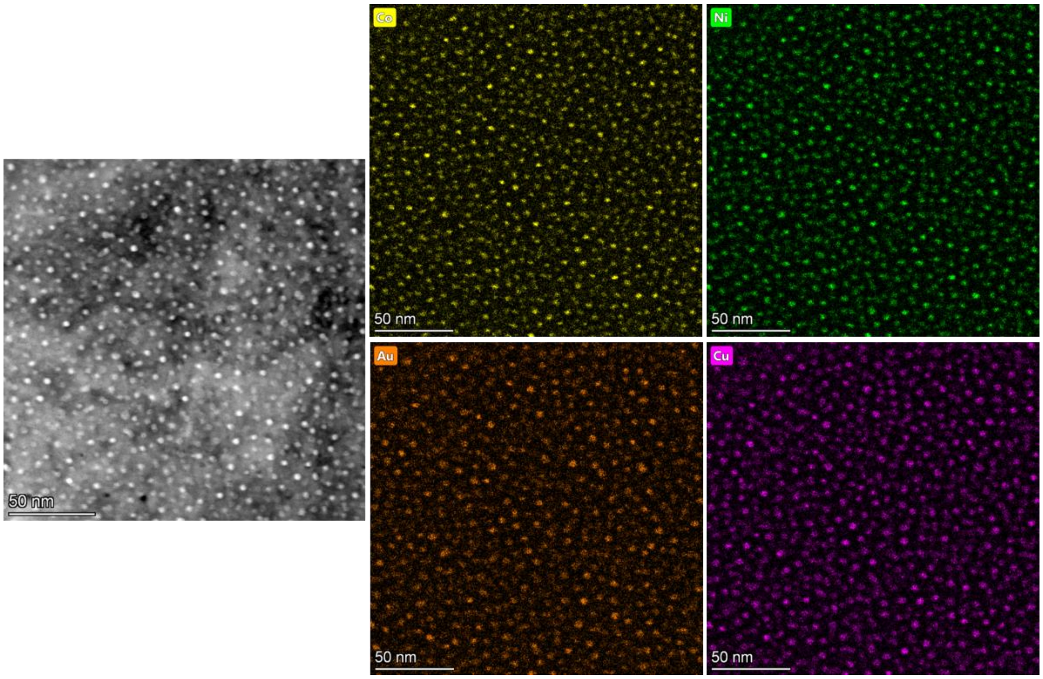

Figure S5: Plan View STEM image of the 600 ^{o}C, 5 Hz alloy sample with elemental mapping of the nanopillars

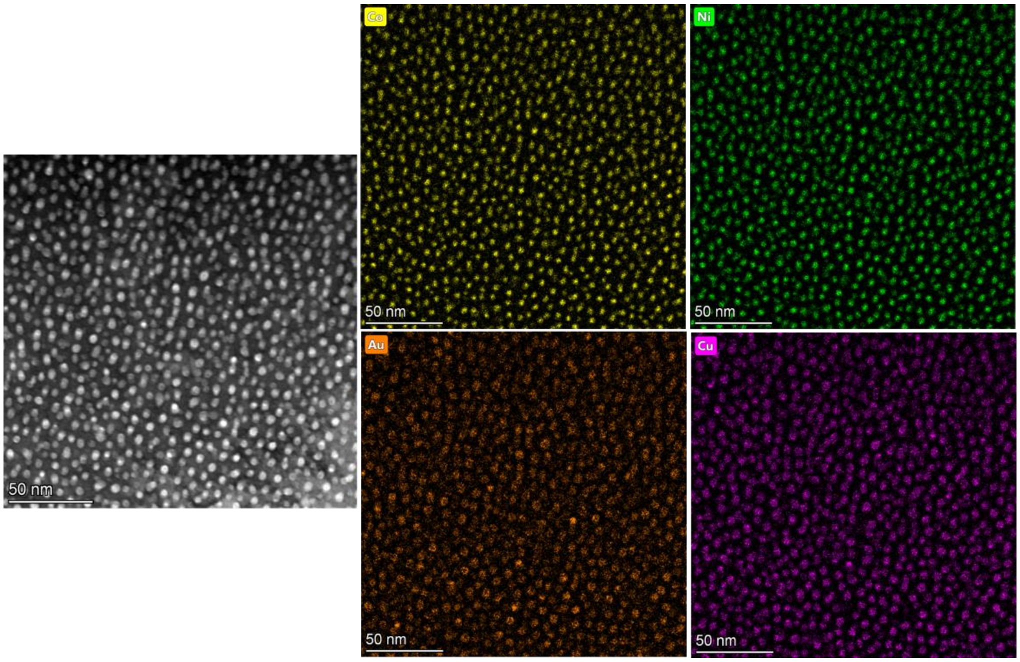

Figure S6: Plan View STEM image of the 600 ^{o}C, 10 Hz alloy sample with elemental mapping of the nanopillars

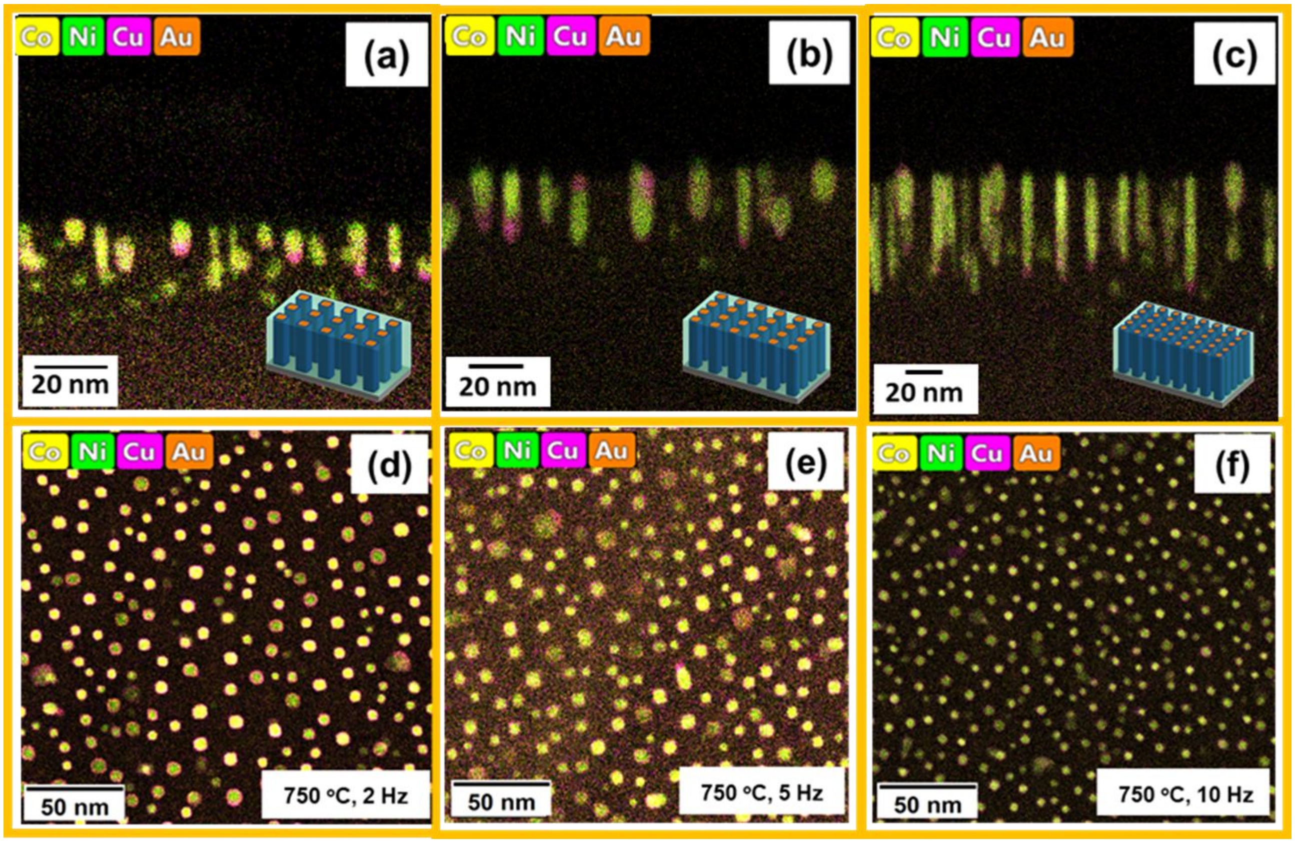

Figure S7 Cross-section EDS profile of all the alloy VAN films deposited at 750 °C with the schematics shown in the inset having deposition frequencies of **(a)** 2 Hz, **(b)** 5 Hz, and **(c)** 10 Hz. The corresponding plan view images of the same temperature are shown in **(d)** 2 Hz, **(e)** 5 Hz, and **(f)** 10 Hz

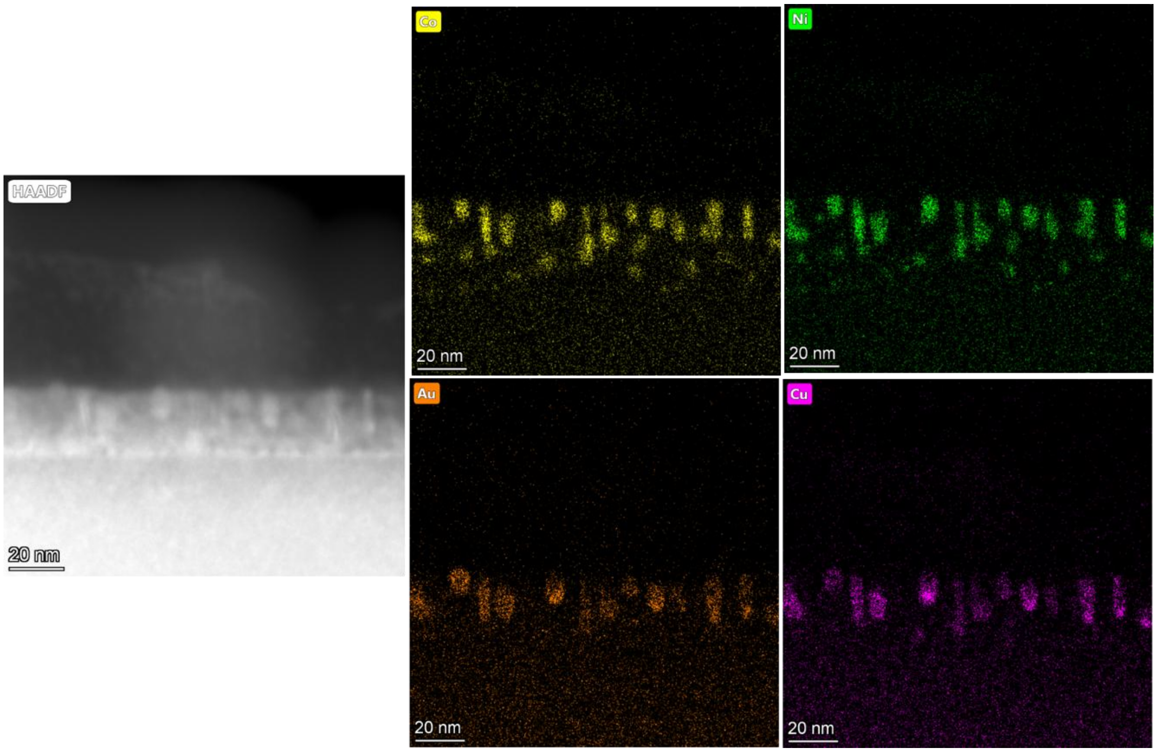

Figure S8: Cross section STEM image of the 750 °C, 2 Hz alloy sample with elemental mapping of the nanopillars

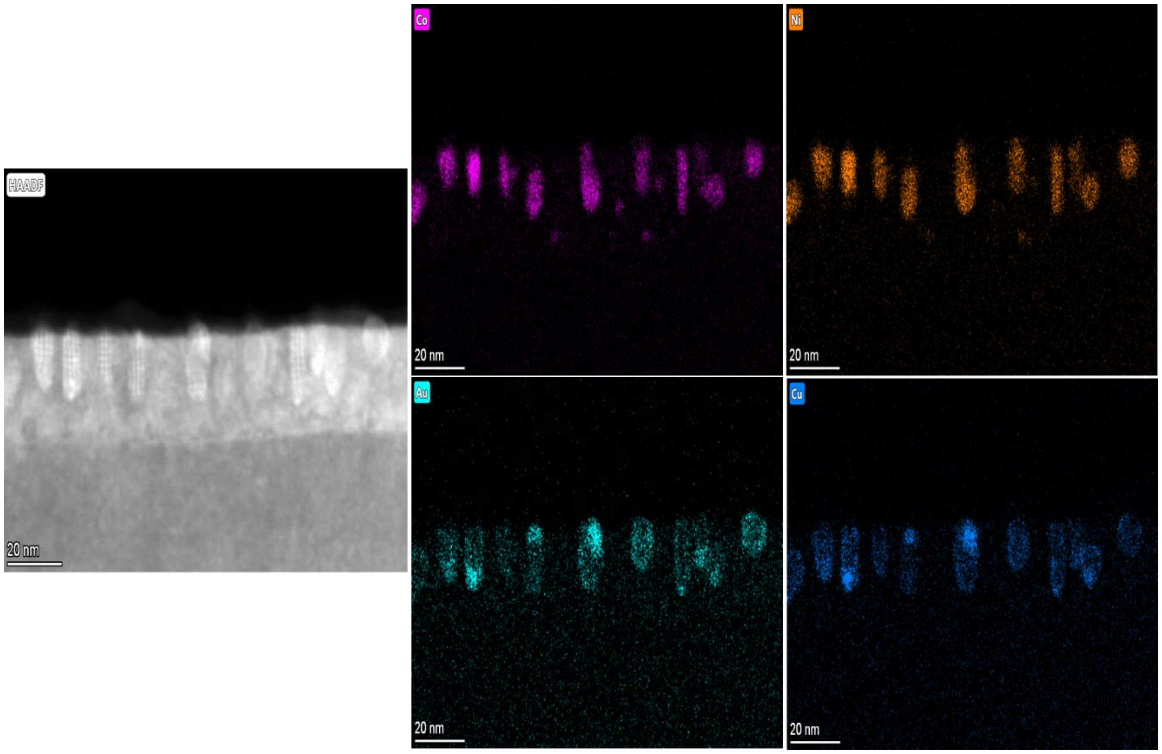

Figure S9: Cross section STEM image of the 750 °C, 5 Hz alloy sample with elemental mapping of the nanopillars

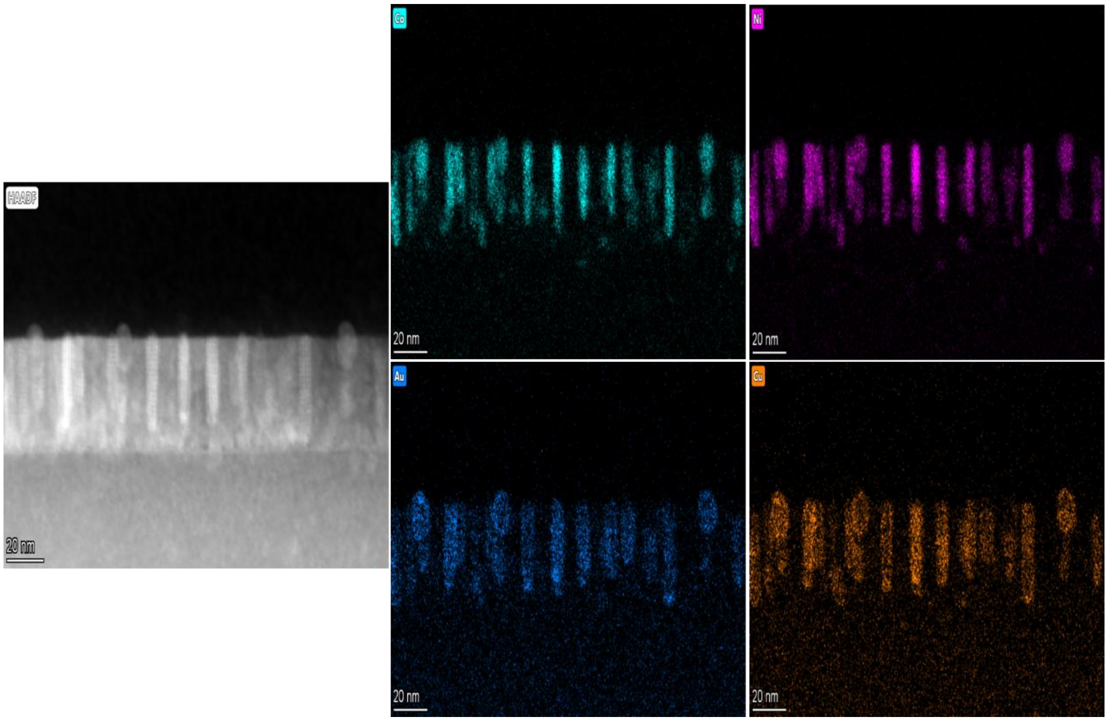

Figure S10: Cross section STEM image of the 750 ^{o}C, 10 Hz alloy sample with elemental mapping of the nanopillars

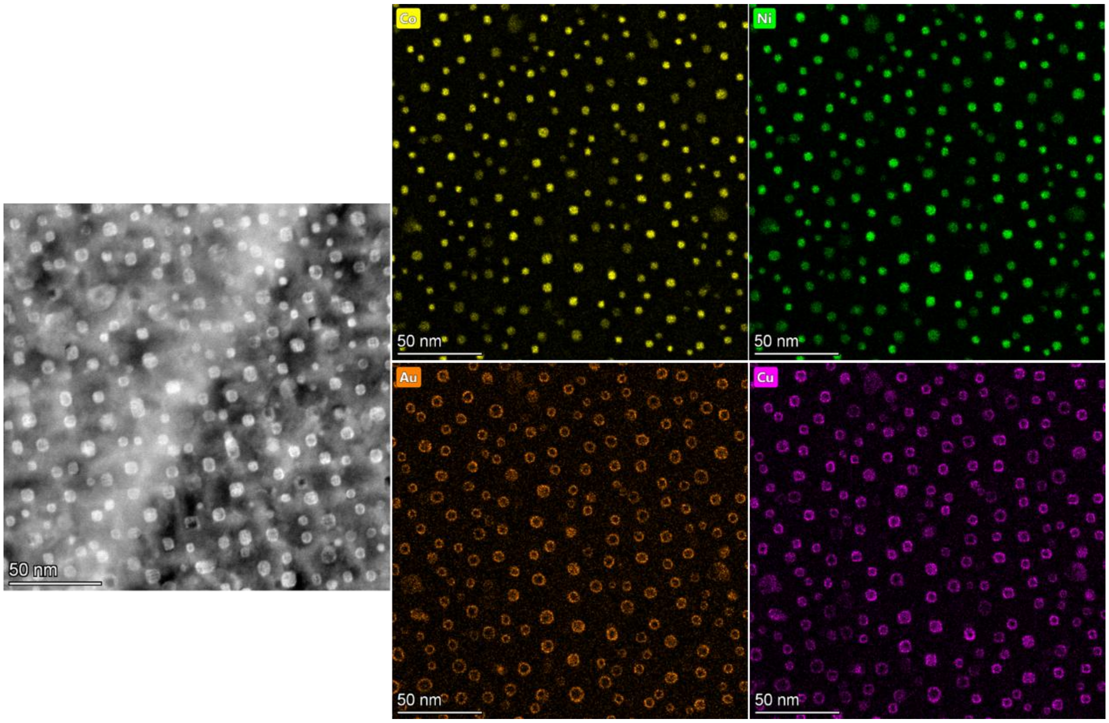

Figure S11: Plan View STEM image of the 750 ^{o}C, 2 Hz alloy sample with elemental mapping of the nanopillars

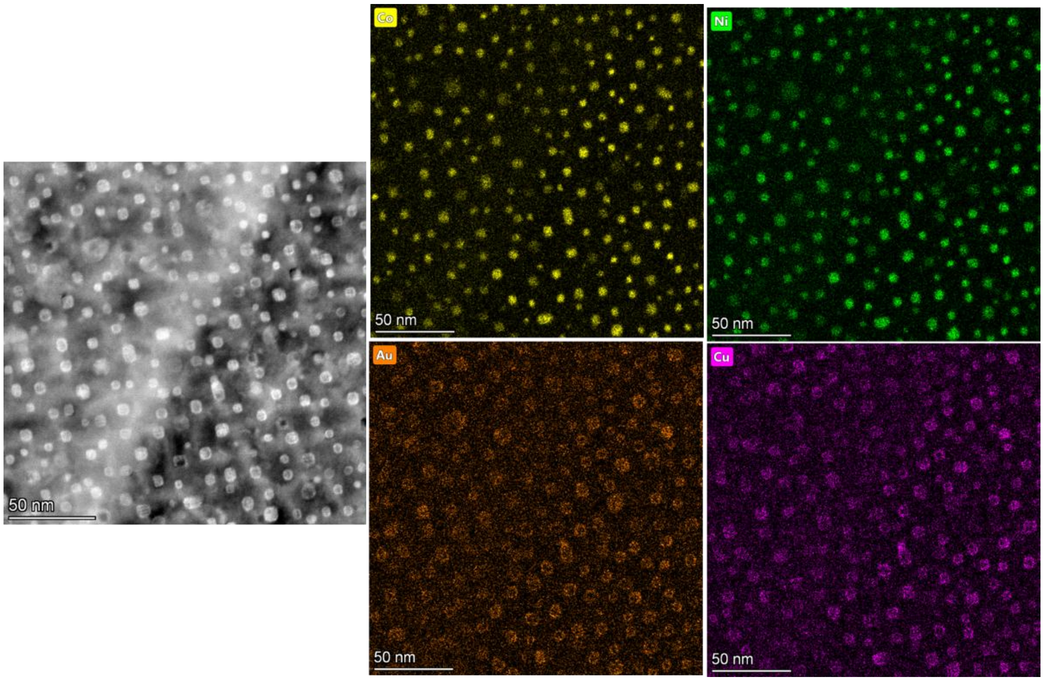

Figure S12: Plan View STEM image of the 750 ^{o}C, 5 Hz alloy sample with elemental mapping of the nanopillars

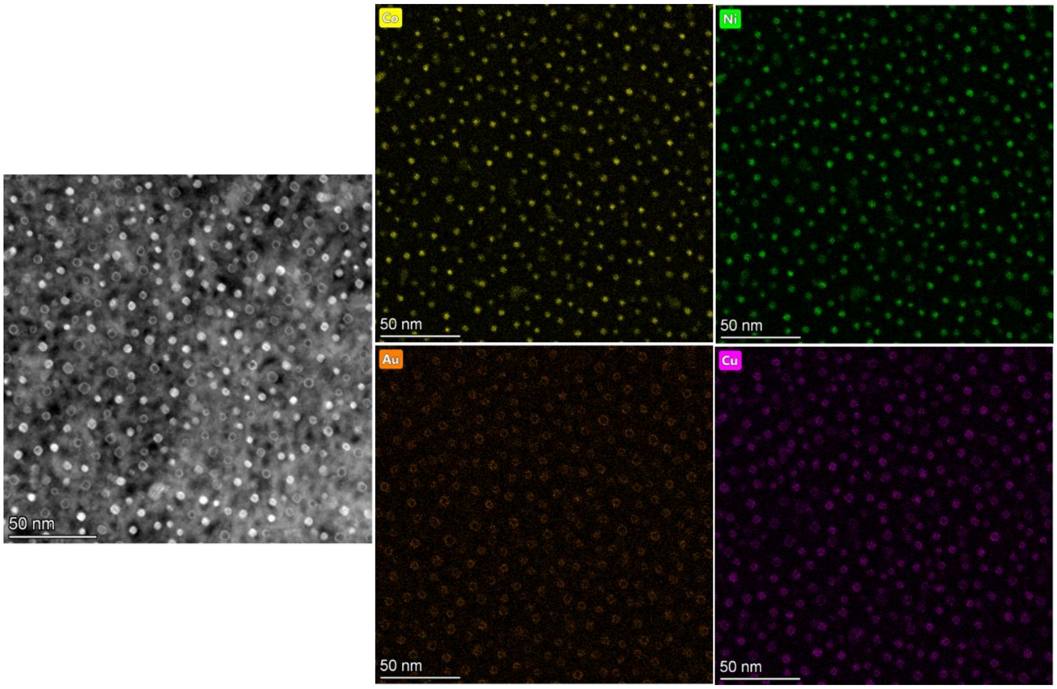

Figure S13: Plan View STEM image of the 750 ^{o}C, 10 Hz alloy sample with elemental mapping of the nanopillars

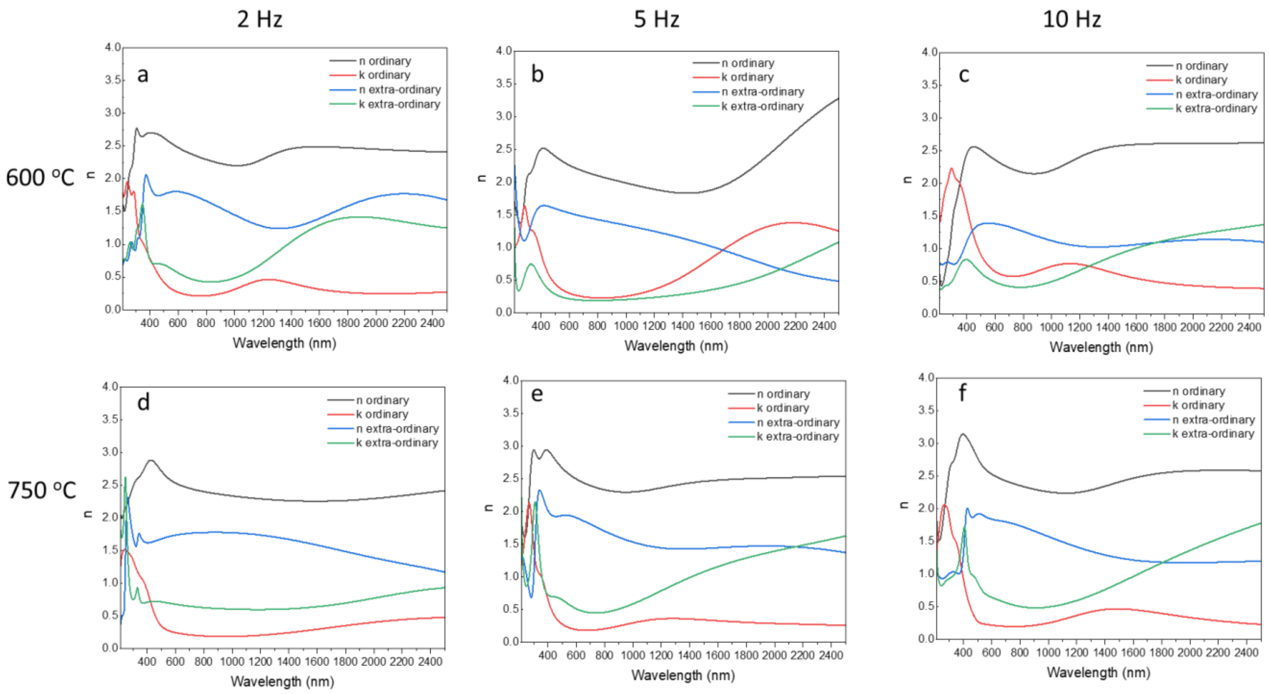

Figure S14: Refractive index (n) and extinction coefficient (k) of all the alloy VAN films. The films were deposited at 600 °C with laser frequencies of **(a)** 2 Hz, **(b)** 5 Hz, and **(c)** 10 Hz, and at 750 °C with frequencies of **(d)** 2 Hz, **(e)** 5 Hz, and **(f)** 10 Hz

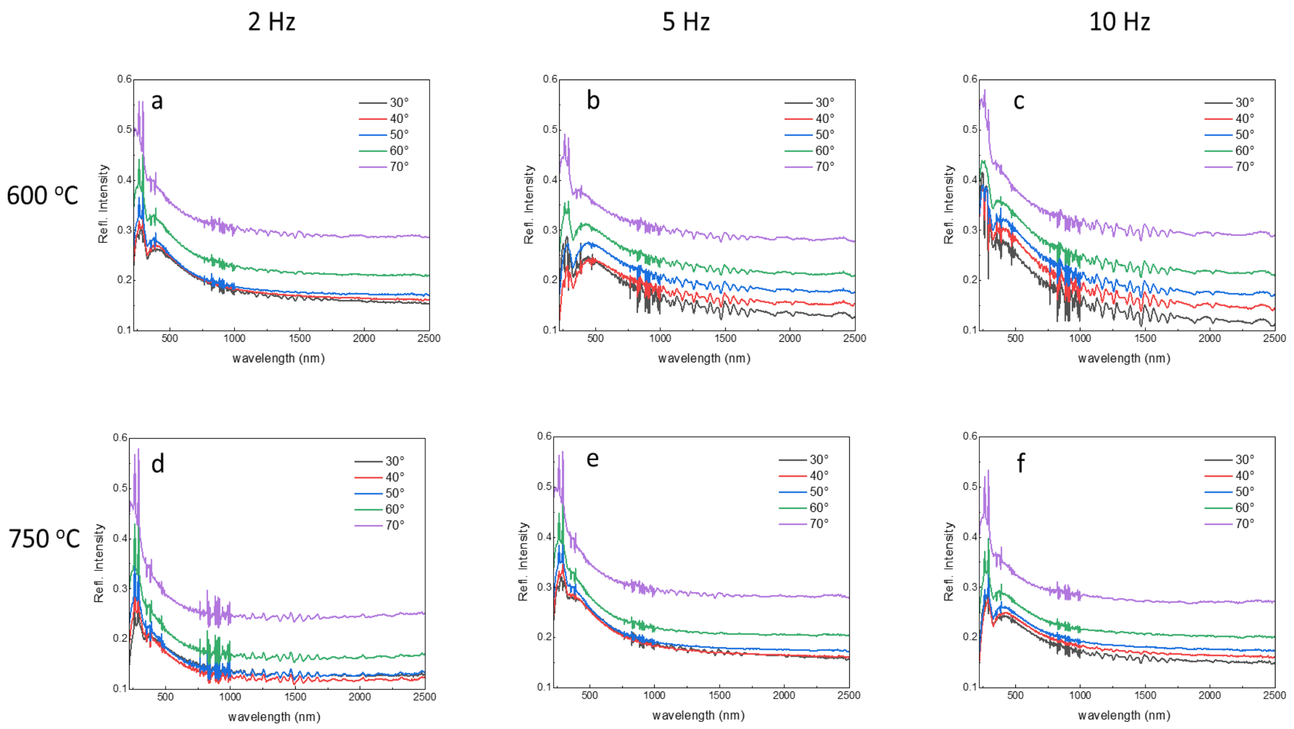

Figure S15: Reflection Intensities at different angles of all the alloy VAN films. The films were deposited at 600 °C with laser frequencies of **(a)** 2 Hz, **(b)** 5 Hz, and **(c)** 10 Hz, and at 750 °C with frequencies of **(d)** 2 Hz, **(e)** 5 Hz, and **(f)** 10 Hz

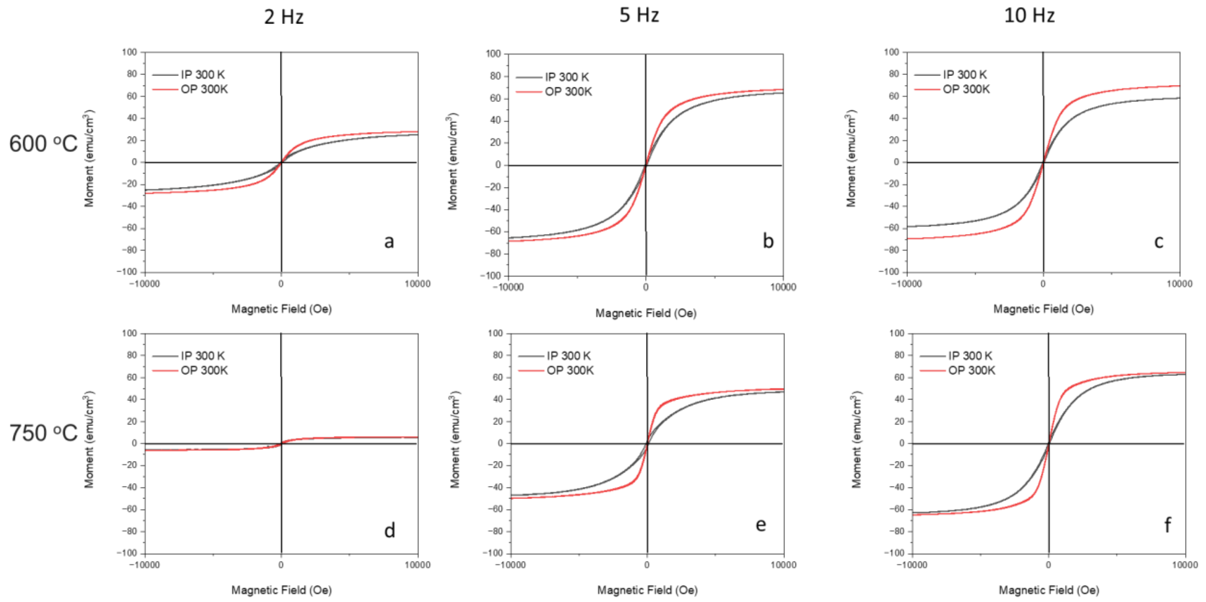

Figure S16: Magnetic hysteresis loops of the Alloy VAN film deposited at 600 °C with frequencies of **(a)** 2 Hz, **(b)** 5 Hz, and **(c)** 10 Hz, and at 750 °C with frequencies of **(d)** 2 Hz, **(e)** 5 Hz, and **(f)** 10 Hz at 300 K

Material	Surface Energy (J/m^2)
STO (001)	1.26 [33]
BTO (001)	1.24 [33]
Au (001)	1.627 [31]
Cu (001)	1.802 [31]
Co (001)	3.19 [32]
Ni (001)	2.426 [31]

Table S3: Surface energies of all the materials in the film

Sample	Mean Diameter (in nm)	Standard Deviation (in nm)
600 °C, 2 Hz	6.5	0.8
600 °C, 5 Hz	5.6	0.7
600 °C, 10 Hz	4.6	0.7
750 °C, 2 Hz	6.3	0.6
750 °C, 5 Hz	5.8	0.6
750 °C, 10 Hz	4.5	0.7

Table S4: Pillar Diameters of all the films calculated based on plan-view STEM images.

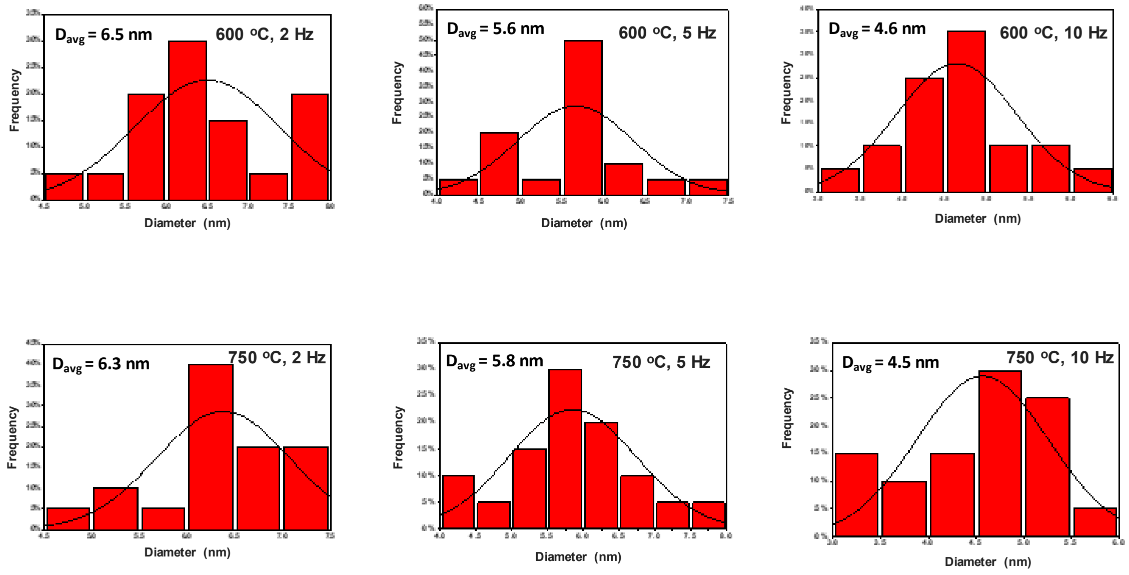

Figure S17: Diameter histograms of nanopillars measured from plan-view TEM for alloy VAN films

Film	BTO out-of-plane parameter (Å)	BTO in-plane parameter (Å)
Pure BTO grown in vacuum	4.077	3.974
600 °C, 2 Hz	4.080	3.973
600 °C, 5 Hz	4.104	3.961
600 °C, 10 Hz	4.081	3.972
750 °C, 2 Hz	4.083	3.971
750 °C, 5 Hz	4.088	3.969
750 °C, 10 Hz	4.084	3.971

Table S5: BTO lattice parameters calculated

Sample	FWHM$_x$
600 °C, 2 Hz	0.0289^o
600 °C, 5 Hz	0.0122^o
600 °C, 10 Hz	0.0162^o
750 °C, 2 Hz	0.0096^o
750 °C, 5 Hz	0.0064^o
750 °C, 10 Hz	0.0252^o

Table S5: FWHMx values of the films

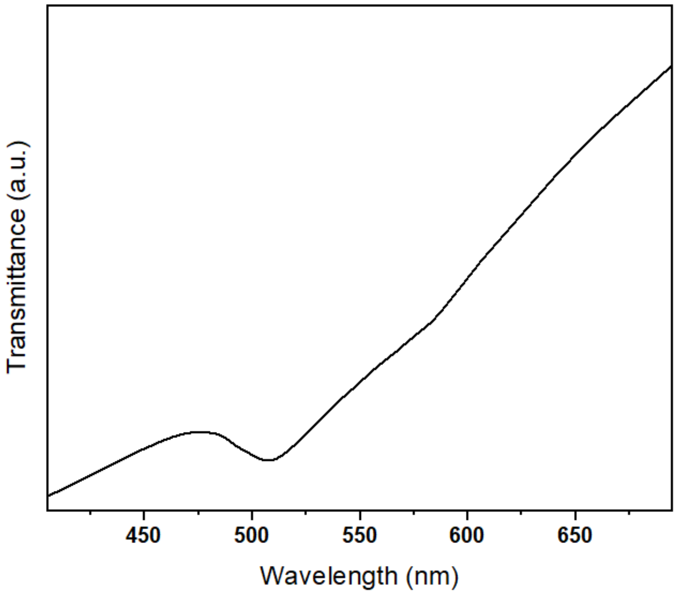

Figure S17: Simulated Transmittance spectra from COMSOL model

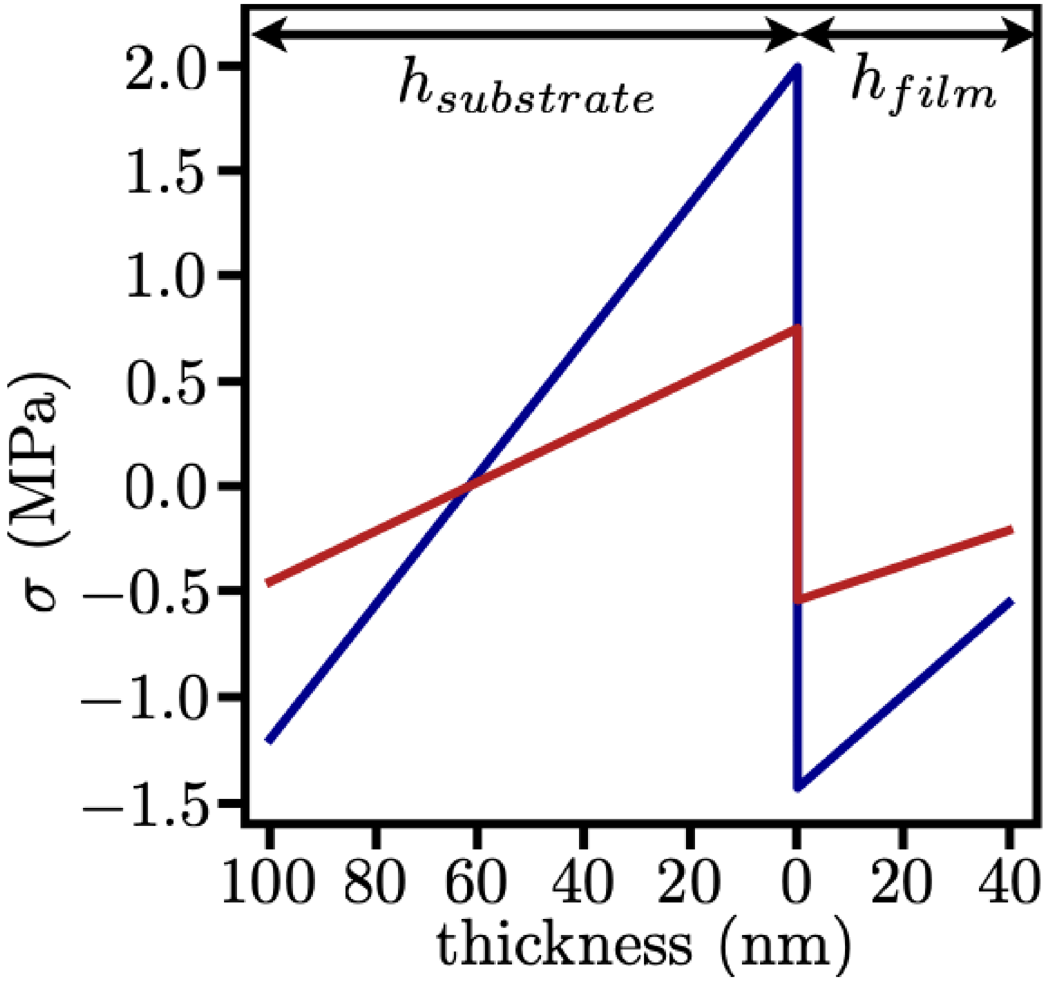

Figure S18: The stress profile of a bilayer due to the epitaxial and thermal expansion coefficient mismatch between the substrate and the pillar-matrix film. ▬ corresponds to the stress in the bilayer at $T = 873K$, and ▬ at $T = 1023K$. The interplay between the thermal and epitaxial stresses results in lower compression stress state inside the pillar-matrix film, which in turn determines the equilibrium shape of the pillar.

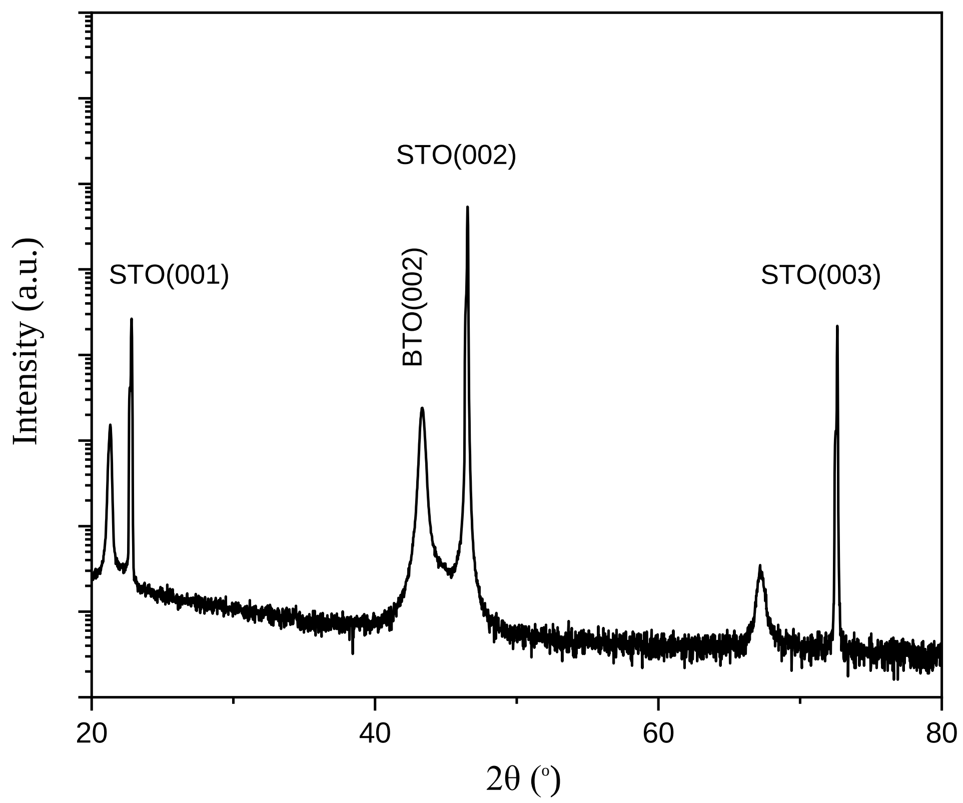

Figure S19: ***(a)*** *θ-2θ* XRD scans of pure BTO film grown and cooled in Vacuum (750 °C, 10 Hz) on STO substrate

[1] O. Redlich and A. T. Kister, "Algebraic Representation of Thermodynamic Properties and the Classification of Solutions.," *Industrial and Engineering Chemistry,* vol. 40, pp. 345-348, 1948.

[2] J. W. Cahn and C. A. Handwerker, "Equilibrium Geometries of Anisotropic Surfaces and Interfaces.," *Materials Science and Engineering,* vol. A162L, pp. 83-95, 1993.

[3] J. E. Blendel and a. C. A. H. W. C. Carter, "Faceting and Wetting Transitions of Anisotropic Interfaces and Grain Boundaries.," *Journal of the American Ceramic Society,* vol. 82, no. 7, pp. 1889-1900, 1999.

[4] G. Wulff, "Zur Frage der Geschwindigkeit des Wachsthumsund der Auflösung der Krystallflächen.," *Zeitschrift für Kristallographie,* vol. 34, p. 449, 1901.

[5] J. Lund, H. Wang, R. D. Braatz and R. E. García, "Machine Learning of Phase Diagrams.," *Materials Advances,* vol. 3, pp. 8485-8497, 2022.

[6] J. Kubišta and J. Vreštál, "Thermodynamics of the Liquid Co-Cu System and Calculation of Phase Diagram.," *Journal of Phase Equilibria,* vol. 20, no. 2, pp. 125-129, 2000.

[7] J. Huang, B. Zhang, D. Hermawan, A. Sanjuan, B. K. Tsai, J. Huang, R. E. García and H. Wang, "Complex Oxide-Metal Hybrid Metamaterials with Integrated Magnetic and Plasmonic Non-Noble Metal Nanostructures.," *Advanced Functional Materials,* vol. 35, p. 2500741, 2025.

[8] J. E. Guyer, D. Wheeler and J. A. Warren, "FiPy: Partial Differential Equations with Python.," *Computing in Science and Engineering,* vol. 11, no. 3, pp. 6-15, 2009.

[9] R. E. García, A. C. E. Reid, S. A. Langer and W. C. Carter, "Microstructural Modeling of Multifunctional Material Properties: The OOF Project," in *Continuum Scale Simulation of Engineering Materials,* New Jersey, Wiley-VCH, 2004..

[10] P. Virtanen, R. Gommers, T. E. Oliphant, M. Haberland, T. Reddy, ,. D. Cournapeau, E. Burovski, P. Peterson, W. Weckesser, J. Bright, S. J. van der Walt, M. Brett, J. Wilson, K. J. Millman, N. Mayorov, A. R. J. Nelson, E. Jones, R. Kern and L, "SciPy 1.0: Fundamental Algorithms for Scientific Computing in Python.," *Nature Methods,* vol. 17, pp. 261-272, 2020.

[11] C. R. Harris, K. J. Millman, S. J. van der Walt, R. Gommers, P. Virtanen, D. Cournapeau, E. Wieser, J. Taylor, S. Berg, N. J. Smith, R. Kern, M. Picus, S. Hoyer, M. H. van Kerkwijk, M. Brett, A. Haldane, J. Fernández del Río and M. Wiebe, "Array Programming with NumPy," *Nature,* vol. 585, pp. 357-362, 2020.

[12] F. Jona and G. Shirane, Ferroelectric Crystals, New York: Dover Publications, 1993.

[13] The Materials Project, "Materials Data on Cu (SG:225) by Materials Project.," *United States,* 2020.

[14] S. J. Rothman and N. L. Peterson, "Isotope Effect and Divacancies for Self-Diffusion in Copper," *Physica Status Solidi B,* vol. 35, pp. 205-312, 1969.

[15] M. McLean, "Determination of the Surface Energy of Copper as a Function of Crystallographic Orientation and Temperature," *Acta Metallurgica,* vol. 19, pp. 397-393, 1971.

[16] G. C. C. Costa, P. S. Maram and A. Navrotsky, "Thermodynamics of Nanoscale Lead Titanate and Barium Titanate Perovskites.," *Journal of the American Ceramic Society,* vol. 95, no. 10, pp. 3254-3262, 2012.

[17] J.-K. Liou, M.-H. Lin and H.-Y. Liu, "Crystallographic Facetting in Sintered Barium Titanate.," *Journal of the American Ceramic Society,* vol. 85, no. 12, pp. 2931-2937, 2002.

[18] B. Sundman, S. G. Fries and W. A. Oates, "A Thermodynamic Assessment of the Au-Cu System.," *Calphad,* vol. 22, no. 3, pp. 335-354, 1998.

[19] M. A. Turchanin, P. G. Agraval and A. R. Abdulov, "Phase Equilibria and Thermodynamics of Binary Copper Systems with 3d-metals. VI. Copper-Nickel System.," *Powder Metallurgy and Metal Ceramics,* vol. 46, pp. 9-10, 2007.

[20] R. O. Bell and G. Rupprecht, "Elastic Constants of Strontium Titanate," *Physical Review,* vol. 129, no. 1, pp. 90-94, 1963.

[21] J. R. Neighbours and G. A. Alers, "Elastic Constants of Silver and Gold," *Physical Review,* vol. 111, no. 3, pp. 707-711, 1958.

[22] J. Gump, H. C. M. S. R. Xia, M. A. Tomaz and G. R. Harp, "Elastic Constants of Face Centered Cubic Cobalt," *Journal of Applied Physics,* vol. 86, no. 11, pp. 6005-6009, 1999.

[23] H. M. Ledbetter and E. R. Naimon, "Elastic Properties of Metals and Alloys II. Copper," *Physical and Chemical Reference Data,* vol. 3, no. 4, p. 897, 1974.

[24] J. R. Neighbours, F. W. Bratten and C. S. Smith, "The Elastic Constants of Nickel," *Journal of Applied Physics,* vol. 23, pp. 389-393, 1952.

[25] W. R. Cook, D. A. Berlincourt and F. J. Scholtz, "Thermal Expansion and Pyroelectricity in Lead Titanate Zirconate and Barium Titanate," *Journal of Applied Physics,* vol. 34, pp. 1392-1398, 1963.

[26] B. N. Dutta and B. Dayal, "Lattice Constants and Thermal Expansion of Gold up to 878oC by X-Ray Method," *Physica Status Solidi,* vol. 3, pp. 473-477, 1963.

[27] R. N. Abdullaev, R. A. Khairulin, Y. M. Kozlovsky and S. V. Stankus, "Density and Thermal Expansion of High Purity Cobalt over the Temperature Range from 140 to 2073 K," *Metallurgical and Materials Transactions A,* vol. 52A, pp. 5449-5456, 2021.

[28] T. A. Hahn, "Thermal Expansion of Copper from 20 to 800 K - Standard Reference Material 736," *Journal of Applied Physics,* vol. 41, pp. 5096-5101, 1970.

[29] T. G. Kollie, "Measurement of Thermal Expansion Coefficient of Nickel from 300 to 1000 K and Determination of the Power Law Constants Near the Curie Temperature," *Physical Review B,* vol. 16, no. 11, pp. 4872-4881, 1977.

[30] D. de Ligny and P. Richet, "High-Temperature Heat Capacity and Thermal Expansion of SrTiO3 and SrZrO3 Perovskite," *Physical Review B,* vol. 53, no. 6, pp. 3013-3022, 1996.

[31] Vitos, L., Ruban, A. V., Skriver, H. L., & Kollár, J. (1998). The surface energy of metals. Surface science, 411(1-2), 186-202.

[32] Swart, J. C., van Helden, P., & van Steen, E. (2007). Surface energy estimation of catalytically relevant fcc transition metals using DFT calculations on nanorods. *The Journal of Physical Chemistry C*, *111*(13), 4998-5005.

[33] Meyer, B., Padilla, J., & Vanderbilt, D. (1999). Theory of PbTiO3, BaTiO3, and SrTiO3 surfaces. *Faraday Discussions*, *114*, 395-405.
